# Management of Pediatric Urinary Tract Infections: A Delphi Study

**DOI:** 10.3390/antibiotics11081122

**Published:** 2022-08-18

**Authors:** Giovanni Autore, Luca Bernardi, Claudio La Scola, Filippo Ghidini, Federico Marchetti, Andrea Pasini, Luca Pierantoni, Claudia Castellini, Claudia Gatti, Cristina Malaventura, Gabriella Pelusi, Francesco Antodaro, Andrea Bergomi, Franco Mazzini, Giovanni Parente, Roberto Pillon, Francesca Cusenza, Giacomo Biasucci, Alessandro De Fanti, Lorenzo Iughetti, Serafina Perrone, Andrea Pession, Mario Lima, Susanna Esposito, The UTI-Ped-ER Study Group

**Affiliations:** 1 Pediatric Clinic, University Hospital, Department of Medicine and Surgery, University of Parma, 43126 Parma, Italy; 2 Pediatric Clinic, IRCCS Azienda Ospedaliero-Universitaria di Bologna, 40138 Bologna, Italy; 3 Pediatric Surgery, University of Modena and Reggio Emilia, 41125 Modena, Italy; 4 Pediatrics and Neonatology Unit, Ravenna Hospital, AUSL Romagna, 48121 Ravenna, Italy; 5 Pediatric Emergency Unit, IRCCS Azienda Ospedaliero-Universitaria di Bologna, 40138 Bologna, Italy; 6 Pediatric Unit, Carpi Hospital, AUSL Modena, 41012 Carpi, Italy; 7 Pediatric Surgery, University Hospital, 43126 Parma, Italy; 8 Pediatric Clinic, University of Ferrara, 44124 Ferrara, Italy; 9 Pediatrics Surgery, Rimini Hospital, AUSL Romagna, 47921 Rimini, Italy; 10 Primary Care Pediatrician, AUSL Modena, 41125 Modena, Italy; 11 Primary Care Pediatrician, AUSL Romagna, 47521 Cesena, Italy; 12 Pediatric Surgery, IRCCS Azienda Ospedaliero-Universitaria di Bologna, 40138 Bologna, Italy; 13 Pediatric Unit, Maggiore Hospital, 40133 Bologna, Italy; 14 Pediatrics and Neonatology Unit, Guglielmo da Saliceto Hospital, 29122 Piacenza, Italy; 15 Pediatrics Unit, Santa Maria Nuova Hospital, AUSL-IRCCS of Reggio Emilia, 42123 Reggio Emilia, Italy; 16 Pediatrics Unit, Department of Medical and Surgical Sciences of Mothers, Children and Adults, University of Modena and Reggio Emilia, 41125 Modena, Italy; 17 Neonatology Unit, University Hospital, Department of Medicine and Surgery, University of Parma, 43126 Parma, Italy

**Keywords:** antibiotic therapy, antimicrobial resistance, pediatric infectious diseases, pediatric urology, urinary tract infection, urine culture

## Abstract

Urinary tract infection (UTI) is one of the most common infectious diseases in the pediatric population and represents a major cause of antibiotic consumption and hospitalization in children. Considering the ongoing controversies on the management of pediatric UTI and the challenges due to increasing antimicrobial resistance, the aim of the present study was to evaluate the level of agreement on UTI management in pediatric age in Emilia-Romagna Region, Italy, and to assess on the basis of recent studies whether there is the need to change current recommendations used by primary care pediatricians, hospital pediatricians, and pediatric surgeons in everyday clinical practice to possibly improve outcomes. This consensus provides clear and shared indications on UTI management in pediatric age, based on the most updated literature. This work represents, in our opinion, the most complete and up-to-date collection of statements on procedures to follow for pediatric UTI, in order to guide physicians in the management of the patient, standardize approaches, and avoid abuse and misuse of antibiotics. Undoubtedly, more randomized and controlled trials are needed in the pediatric population to better define the best therapeutic management in cases with antimicrobial resistance and real usefulness of long-term antibiotic prophylaxis.

## 1. Introduction

Urinary tract infection (UTI) is one of the most common infectious diseases in pediatric population and represents a major cause of antibiotic consumption and hospitalization in children [1]. UTIs affect up to 2.8% of children annually in high-income countries, with recurrence rates ranging from 8% to 30% [2,3]. It is estimated that 11.3% of females and 3.6% of males develop at least one episode of UTI within the first 16 years of life [4]. The prevalence of UTI has a bimodal trend, with a first peak within the first year of life and a second between 2 and 4 years of age, corresponding to toilet training [5]. During the first 6 months of life, the risk of UTI is greater for males, particularly for uncircumcised boys. The male to female ratio is reversed after the first year of life, with increased risk for females that persists into adulthood [6]. Gram-negative microorganisms of the *Enterobacterales* order are the most common pathogens in pediatric UTIs, with *Escherichia coli* accounting for more than 70% of first and recurrent episodes [7,8].

UTI represents an everyday issue for primary care pediatricians, but also a major cause of pediatric emergency department visits. In the US, up to 0.5% of all children aged under 17 years receive care for UTI in an emergency department setting annually [9]. Therefore, cooperation between primary care and hospital physicians and agreement on common guidelines are essential for the appropriate management of pediatric UTIs. Although several specific and well-established guidelines have been published in past years, the management of pediatric UTI remains controversial. Children with UTIs are often investigated and treated quite differently, depending on countries, clinical settings, and specialization [10].

Diagnosis of pediatric UTI is challenging due to its nonspecific clinical presentation. UTI should be considered in differential diagnosis of children presenting fever with no apparent source [11]. Fever may be the only clinical sign, especially in young children [12]. Newborns and infants younger than 3 months often present with nonspecific signs including feeding difficulties, lethargy or irritability, jaundice, vomiting, and sometimes without fever or with hypothermia [13]. However, the absence of fever at this age does not correlate with a less severe infection, and the risk of complications, such as urosepsis, is greater [14]. Older children may report lower urinary tract symptoms, such as frequency, dysuria, and continence dysfunctions, but also abdominal pain and loin tenderness in association with fever [13]. Upper UTIs involve the renal pelvis and/or kidney (pyelonephritis) [13,14]. Clinically, an upper UTI should be assumed if there is bacteriuria and fever of 38 °C or higher, or if there is a fever lower than 38 °C with loin pain/tenderness and bacteriuria. Lower UTIs involve the bladder and urethra [13,14]. In several cases, UTIs in children are undifferentiated because it is not possible to distinguish between the two conditions above.

Urine sampling is crucial for the diagnosis of UTI, but still presents controversies in daily practice. The technique used to obtain urine for urinalysis or culture affects the rate of contamination that in turn influences interpretation of the results, especially in early infancy. Suprapubic aspiration and bladder catheterization have the lowest contamination rates, but represent invasive procedures. Conversely, a plastic bag attached to genitalia is the most used technique to collect urine in daily practice, but, although simple and noninvasive, bag samples have a rate of contamination up to 63% and should not be used for culture [15]. Clean-catch urine collection is a valid alternative, despite a contamination rate of up to 26% [16]. The role of urine dipstick in the diagnosis of UTI is still debated. When nitrite or both leukocyte esterase and nitrite results are positive, specificity for the diagnosis of UTI is sufficiently high to regard the patient as having a UTI and to start empirical treatment [17]. In these cases, guidelines from the National Institute for Health and Clinical Excellence (NICE) suggest further performing urine culture only in patients at risk of serious illness or with a history of recurrent UTIs [13]. On the other hand, guidelines provided by the Italian Society of Pediatric Nephrology and European Association of Urology (EAU)/European Society on Pediatric Urology (ESPU) strongly recommend to always perform urine culture in cases of positive results at dipstick [18]. When only leukocyte esterase tests positive, the indication to start antibiotic treatment widely depends on clinical suspicion. Recent updates of different guidelines have generally lowered the thresholds for definition of significant positive urine culture, but they still depend on the technique used to obtain urine [13]. The cutoff of 50,000 CFU/mL is generally considered reasonable. However, some guidelines have proposed lower thresholds for urine collected through suprapubic aspiration or catheterization and higher cutoffs for clean-catch and bag samples [18]. An accurate diagnostic approach is necessary, because—even though acute septic complications are uncommon—up to 40% of infections may result in permanent renal scarring [19,20].

UTI is the first and often the only sign in about one third of children with urinary tract anomalies [21]. On the other hand, inappropriate criteria for hospitalization and overuse of diagnostic tools, such as unnecessary imaging, contribute to the rapid increase in UTI-related economic burden that has been reported in recent years [22].

The empirical treatment for pediatric UTI is also a controversial field and most guidelines suggest a wide range of molecules as equally suitable empirical treatments [13,23]. Therefore, the choice of initial antibiotic is often left to personal experience. The misuse and overuse of antibiotics is a leading cause of the alarming spread of antibiotic resistance in community-acquired pediatric UTIs that, in turn, prompted the empirical use of broad-spectrum molecules in a vicious cycle [24]. According to a recent European study, health-care costs more than doubled for children infected by resistant uropathogens, mainly due to the increased length of stay [25]. A recent surveillance study conducted in our region on antimicrobial resistance observed in pediatric UTIs showed an increase in resistance rates and questioned the appropriateness of some first-line treatments, suggesting the need for a differentiated approach based on risk factors of resistant infection [26,27].

Considering the ongoing controversies on the management of pediatric UTI and the challenges due to the increasing antimicrobial resistance, the aim of the present study was to evaluate the level of agreement on UTI management in pediatric age in Emilia-Romagna Region, Italy, and to assess on the basis of recent studies whether there is a need to change current recommendations used by primary care pediatricians, hospital pediatricians, and pediatric surgeons in everyday clinical practice to possibly improve outcomes.

## 2. Methods

A Delphi method was used to reach the abovementioned aims [28]. It represents an indirect, anonymous, iterative process aimed at achieving comprehensive consensus among experts on specific topics, especially regarding disease management and drug therapy. A board of experts in the field of pediatrics and pediatric surgery was appointed as a scientific committee in charge of designing and supervising the study. A panel of experts in the field of primary care pediatrics, pediatric nephrology, and pediatric urologic surgery was thereafter selected by the board on the basis of their skills in research and/or clinical experience. Experts were directors of the pediatric units in hospitals in Emilia-Romagna, pediatricians in charge of the outpatient clinic for pediatric nephrology, surgeons in charge of pediatric urologic surgery in the hospitals of the region, and primary care pediatricians representing each regional capital. In addition, a microbiologist and a clinical pharmacologist were included. Local epidemiology was considered, but the aim was to develop a consensus document with the ambition to assess whether the Italian guidelines on the management of UTI need to be changed, making a contribution also at European and extra-European level.

A questionnaire with 81 questions was developed by the project coordinators and the scientific committee (Supplementary Materials S1 Questions covered six main topics identified on the basis of clinical experience and available literature: (1) epidemiology (burden of disease, etiology and antimicrobial resistance); (2) diagnosis (clinical findings, urine dipstick, urine sampling); (3) management (hospitalization, indication for exams); (4) therapy (first-line empirical therapy, second-line therapy, duration of treatment); (5) imaging (echography, cystography, cystosonography, scintigraphy); and (6) prophylaxis (indication, drug of choice). The questions were proposed after a careful revision of current scientific literature, including original research, reviews and systematic reviews, meta-analysis, recommendations, and guidelines, selected using Medline and PubMed. For every question, the literature including its revision was provided to the expert panel.

According to the Delphi method, questions with statements were uploaded onto a dedicated online platform to be voted on by the panel of experts using a 5-point Likert scale (from 1 = complete disagreement to 5 = complete agreement). The first round of the survey was blind to other panel members. The experts responded through the online survey application Google Forms within a 1-month deadline. Round 1 responses were collected and sent to an independent statistician for analysis. Results of the survey were discussed in a kickoff meeting, during which the answers to the first survey and indications were shown. Clarifications, adaptations, and refinements of the indications and appropriateness ratings were made.

According to the most frequently adopted Delphi method, the definition of consensus was set to at least 75% agreement for 4 + 5 scores. After reaching an agreement ≥ 75% for all recommendations, all the authors reviewed and approved the final manuscript and Supplementary Materials. Overall, 21 statements were developed from the 81 questions. The data analysis was performed with Stata 11 (StataCorp, College Station, TX, USA). Microsoft Excel was used for graphic data processing and presentation. Results are presented in bar and pie charts, as shown in Supplementary Materials S2.

## 3. Results

### 3.1. Epidemiology

**Statement** **1.** *E. coli is the most common pathogen in pediatric UTIs (i.e., patients < 18 years old) accounting for more than 70% of all cases, followed by Klebsiella spp., Enterobacter spp., and Proteus spp. Pseudomonas aeruginosa is uncommon in community-acquired pediatric UTIs but it is associated with more severe infections. Up to 30% of pediatric patients experience a recurrence after the first episode of UTI*.

Gram-negative bacteria of the Enterobacterales order represent the most common etiologies of UTIs in pediatric patients [29]. *E coli* is by far the most frequently isolated pathogen, regardless of sex, age, and ethnicity, accounting for up to 80–90% of cases [30]. A comparative study conducted on newborns, infants, and young children aged under 24 months observed a predominance of *E. coli* in all age groups, both in males and females, with a mean prevalence of 72.6%, followed by *Klebsiella* spp. and *Proteus* spp. [31]. In the same age-groups, Hsu et al. observed higher prevalence of *E. coli* that accounted for 86.3% of cases [32].

Few studies are available on patients aged under 3 months. Segal et al. reported that *E. coli* was isolated in 76.4% of UTIs that occurred in infants younger than 2 months, followed by *Klebsiella pneumoniae* and *Enterobacter cloacae* in 13.8% and 1.9% of cases, respectively [33]. *Pseudomonas aeruginosa* was isolated only in 0.9% of infants. * E. coli* was the most frequently isolated uropathogen also in older patients up to 18 years old, both in outpatient and inpatient settings [34]. However, prevalence of *E. coli* seems lower in hospitalized patients, underling the role of different uropathogens in severe UTIs.

Infections caused by non-*E. coli* pathogens (i.e., *K. pneumoniae*, *P. aeruginosa* and *Proteus mirabilis*) are more frequent in younger males [31]. Recognized risk factors for non-*E. coli* UTI infections are recurrent UTIs, due to previous treatment with several antibiotic courses, invasive procedures or indwelling catheters [35]. Among them, *P. aeruginosa* is associated with more severe infections [36,37].

Overall, all the text in Statement 1 obtained sufficient agreement during the first survey and was confirmed in the second survey.

**Statement** **2.** *Prevalence of pathogens resistant to antibiotic therapy varies widely in different geographical areas. The main risk factors for UTIs caused by resistant pathogens include urinary tract anatomical or functional abnormalities, long-term antibiotic prophylaxis, and exposure to antibiotics during the previous 30 days*.

Despite differences among studies, the most reported predisposing factors for the development of resistant febrile UTI were the presence of urinary system structural or functional abnormalities, including vesicoureteral reflux (VUR), recurrent UTIs, and administration of continuous antibiotic prophylaxis [36,37]. A systematic review and meta-analysis reported that compared to children without risk factors, the odds ratio (OR) for the development of UTIs caused by extended-spectrum beta-lactamase (ESBL)-producing *Enterobacteriaceae* was 2.79 (95% confidence interval [CI] 1.39–5.58; *p* = 0.004) in children with VUR and 2.89 (95% CI 1.78–4.68; *p* < 0.001) in children with recurrent UTIs [38]. An observational retrospective study reported that a history of recurrent UTI, antibiotic prophylaxis, and antibiotic therapy in the preceding 30 days was significantly associated with an increased risk of UTI due to antimicrobial-resistant, ESBL-producing, extensively drug-resistant (XDR), or multidrug-resistant (MDR) pathogens, whereas urological malformations were significantly associated with a risk of simple patterns of antimicrobial resistance as well as with XDR/MDR UTI [26]. A systematic review and meta-analysis including 77,783 *E. coli* isolates observed that children previously exposed to antibiotics were more likely to have an infection due to resistant strains (OR 13.23, 95% CI 7.84–22.31) [39].

Overall, all the text in Statement 2 obtained sufficient agreement during the first survey and was confirmed in the second survey.

**Statement** **3.** *Prevalence of uropathogens resistant to combinations of penicillins and beta-lactamase inhibitors and third generation cephalosporins in pediatric population is increasing worldwide. Prevalence of ESBL-producing pathogens in pediatric UTIs is globally increasing. Prevalence of MDR pathogens is low but increasing, whereas prevalence of extensively drug-resistant (XDR) pathogens is still low and stable*.

The incidence of uropathogen resistance to commonly used antibiotics for pediatric UTI has increased worldwide. A retrospective study conducted in the USA analyzed 368,398 isolates from children with UTI between January 1999 and December 2011, reporting that 1.97% were resistant to third-generation cephalosporins and 0.47% were identified as ESBL producers. Prevalence rates of both phenotypes increased from 1.39% and 0.28% in 1999–2001 to 3% and 0.92% in 2010–2011, respectively [40]. Similar results were confirmed by a British study, showing that the monthly incidence of ESBL-producing pathogens among pediatric UTIs increased from 9.5% in 2014 to 13.5% in 2015 [41]. In an Italian population of hospitalized children with febrile UTI, resistance to amoxicillin/clavulanic acid was observed in 33.8% of cases between 1 January 2012 and 30 June 2020 in Emilia-Romagna [26]. Infections caused by MDR or XDR pathogens represented only 6.7% and 0.2% of cases, respectively [26]. Similar findings were confirmed by other studies conducted in Europe and the USA [42,43,44]. Differently from prevalence of XDR uropathogens, prevalence of MDR bacteria among children affected by UTI seems low but increasing [45].

Overall, most of the text in Statement 3 obtained sufficient agreement during the first survey. Uncertainty was initially observed about the prevalence of XDR uropathogens in pediatric UTI. After review of recent literature and collective discussion on available epidemiological data, panelists agreed on defining the prevalence of XDR pathogens as low and stable.

### 3.2. Diagnosis

**Statement** **4.** *Diagnosis of UTI should be considered in all children with fever (CT > 38 °C) without clear localization. In children aged <3 months, an episode of UTI may occur with vomiting, irritability or lethargy even without a fever. Lack of fever in children aged <3 months does not correlate with the severity of UTI. The most frequent symptoms in older children with UTI are dysuria, urinary urgency, increased voiding frequency, new-onset incontinence, abdominal pain and low back pain. The detection of malodorous urine is not specific enough to diagnose UTI*.

The analysis of the most recent guidelines shows that UTI in children is difficult to diagnose, mainly in children under 2 years of age, because symptoms and signs are nonspecific [46]. A technical report from the American Academy of Pediatrics estimated that about 5% of children in the first 2 years of life with fever of unknown origin have a UTI [47]. Most guidelines agree that diagnosis of UTI should be considered in all children presenting with fever ≥ 38 °C with no apparent source [11,13,46]. Fever may be the only symptom of UTI, although in children aged 2 to 3 months fever can be absent and vomiting, irritability or lethargy may be the only symptoms [48].

Especially during the first 3 months of life, lack of fever does not correlate with less severe manifestation [49,50,51]. In a retrospective study, Hernandez-Bou et al. observed no differences in rates of bacteremia between febrile and afebrile infants [14]. In older children dysuria, urgency and increased voiding frequency, new-onset incontinence, and abdominal, low-back, and suprapubic pain are the most frequent symptoms of UTI [13].

Parental reporting of malodorous urine increases the probability of diagnosis among young children being evaluated for suspected UTI. However, this association is not strong enough to definitely rule in or out a diagnosis of UTI. In fact, as reported by Gauthier et al. in a prospective consecutive cohort study, the presence of malodorous urine is neither specific nor sensitive enough to help in the diagnosis of UTI [52].

Overall, all the text in Statement 4 obtained sufficient agreement during the first survey and was confirmed in the second survey.

**Statement** **5.** *Rapid extemporaneous urinalysis (dipstick) is indicated in all children with fever (CT > 38 °C) without clear localization and in those that have symptoms and clinical signs compatible with UTI. The presence of leukocyte esterase and nitrite combined shows elevated sensitivity and specificity for the diagnosis of UTI. Isolated presence of nitrites has high specificity but low sensitivity for diagnosis of UTI. Isolated presence of leukocyte esterase has high sensitivity but low specificity for diagnosis of UTI. Absence of nitrites and leukocyte esterase makes diagnosis of UTI highly unlikely. The presence of bacteriuria and leukocyte on microscopic examination of urine on extemporaneous urinalysis is associated with high specificity and sensitivity for diagnosis of UTI. Detection of bacteria in urine alone (i.e., asymptomatic bacteriuria) is not enough for diagnosis of UTI*.

Most guidelines agree that rapid urine dipstick should be performed not only in children with typical UTI symptoms but also in cases of unexplained fever [46]. Williams et al. studied the absolute and relative accuracy of rapid urine tests in a meta-analysis in which it was concluded that a dipstick should be interpreted as positive if either leukocyte esterase or nitrite is positive [17]. Analyzing 95 studies including 95,703 cases, authors reported that sensitivity and specificity for leukocyte esterase were 79% (range 73–84%) and 87% (range 80–92%), for nitrite were 49% (range 41–57%) and 98% (range 96–99%), for both leukocyte esterase and nitrite were 45% (range 30–61%) and 98% (96–99%) [17].

Thresholds of significance for microscopy results widely vary. Most studies consider reliable any presence of microorganisms per high-resolution field. Cutoffs for leukocyturia range from 10 to 2500 cells/µL. Bacteriuria is the most reliable laboratory parameter for the diagnosis of UTI [17].

Leukocyturia alone provides not enough specificity for the diagnosis of UTI [13].

Overall, most of the text in Statement 5 obtained sufficient agreement during the first survey. Initial statements on interpretation of urine extemporaneous microscopic analysis failed to reach sufficient consensus. After collective discussion on available data from literature, the diagnostic role of leukocyturia alone was questioned, while panelists agreed on the high sensitivity and specificity associated with detection of both bacteriuria and leukocyturia.

**Statement** **6.** *Positive urine culture is necessary to confirm the diagnosis of UTI. Urine culture with antibiogram is indicated in case of positive nitrite and/or leukocyte esterase or leukocyturia and bacteriuria. Urine culture is not indicated in absence of both nitrite and leukocyte esterase*.

According to the meta-analysis conducted by Williams et al., guidelines state UTI “very likely” when both leukocyte esterase and nitrite are positive at dipstick and UTI “likely” when only nitrite is positive because of the very high sensitivity and positive predictive value. In these cases, it is suggested to begin antibiotic therapy empirically and to perform urine culture [13,18].

Leukocyte esterase alone presents high sensitivity and negative predictive value. For this reason, NICE guidelines suggest collecting urine samples for microscopy and culture and considering antibiotic treatment only if good clinical evidence of UTI occurs [13]. A similar approach may be adopted at microscopic sediment urinalysis, considering leukocyturia having the same role of leukocyte esterase [13]. Overall, all the text included in Statement 6 obtained sufficient agreement during the first survey and was confirmed in the second survey.

**Statement** **7.** *A urine sample suitable for culture should always be collected before starting empirical antibiotic therapy. The collection of samples for urine culture in children in good clinical condition should be performed by clean catch mid-stream void or transurethral bladder catheterization; for those in compromised general conditions should be performed by transurethral bladder catheterization. The use of a sterile bag for the collection of urine samples for culture may be acceptable only if the bag is placed for less than 20 min and considering significant only bacterial growth >100,000 CFU/mL. Urine culture should be regarded as positive only if a single pathogenic specie is isolated*.

In a febrile child in poor general clinical condition or in a severely ill-appearing child, urine must be collected by transurethral bladder catheterization or suprapubic puncture [13]. Guidelines from the American Academy of Pediatrics (AAP), which are limited to patients aged <2 years, suggest always preferring invasive methods of collection for urine culture because of unacceptable rates of contamination observed with other techniques [23]. However, in children in good clinical condition, a “two-step” approach is feasible, collecting a first sample of urine for dipstick by clean catch midstream void or bag [18,53,54,55]. If dipstick results suggest a diagnosis of UTI, a urine sample for culture should be collected by clean catch midstream void, or catheterization [18,53,54,55].

Despite the lack of strong evidence, a urobag is still considered useful by some authors to collect samples for urine culture if the bag is placed as quickly as possible and for no more than 20 min [56]. In these cases, a higher cutoff of significance should be used when interpreting urine culture growth. Italian and Swiss guidelines suggest a threshold > 100,000 CFU/mL [18,54,56].

Overall, most of the text in Statement 7 obtained sufficient agreement during the first survey. The use of a bag to collect urine samples for culture required further discussion. Agreement was achieved in the second survey, introducing two limitations on the use of this method: the urobag should be in place for as little time as possible (<20 min) and higher thresholds should be used when interpreting urine culture results (>100,000 CFU/mL).

### 3.3. Management

**Statement** **8.** *Hospitalization is suggested for patients aged under 3 months, critically ill children requiring intravenous therapy (i.e., vomiting, dehydration, sepsis), failure of oral therapy (i.e., persistence of fever after 72 h of adequate antibiotics), or poor compliance to oral therapy*.

Hospitalization and intravenous therapy are not always required for pediatric patients affected by febrile UTI. The noninferiority of entirely oral antibiotic regimens has been shown by different clinical trials [57,58]. A multicenter randomized controlled trial conducted on 502 children affected by acute pyelonephritis showed no significant differences between entirely oral and intravenous/oral regimens, in terms of renal scarring and time to defervescence [57]. Neuhaus et al. reported similar findings in a randomized trial comparing oral and intravenous/oral regimens based on cephalosporins [58]. A recent Cochrane systematic review proved that oral antibiotics alone are as effective as a short course (3–4 days) of intravenous antibiotics followed by oral therapy for a total duration of 10–14 days. Moreover, a short course (2–4 days) of intravenous therapy followed by oral treatment is as effective as a longer course (7–10 days) of intravenous therapy alone [59].

Due to the increased risk of urosepsis, hospitalization and intravenous therapy are generally suggested for patients aged under 3 months [13,60]. Moreover, life-threatening hyponatremia and hyperkalemia due to pseudohypoaldosteronism may occur in infants < 2 months of age [61]. In older patients, hospitalization is indicated when intravenous therapy is required, particularly in critically ill children and when urosepsis is clinically suspected [13,18]. Children with dehydration and inability to take antibiotics by mouth need intravenous therapy [60].

Persistence of fever after 72 h of oral antibiotic therapy should be regarded as a treatment failure [60]. In these cases, hospitalization should be considered in order to exclude clinical complications, to reconsider antibiotic therapy on the basis of the susceptibility test, and to evaluate the patient’s compliance with therapy [60].

Overall, all the text included in Statement 8 obtained sufficient agreement during the first survey and was confirmed in the second survey.

**Statement** **9.** *Blood tests are not routinely needed in children affected by febrile UTI. Complete and differential blood count, C-reactive protein (CRP), procalcitonin, and kidney function tests are recommended in children aged under 3 months and in all cases requiring hospitalization*.

Blood tests are not required for the diagnosis of UTI in well-appearing children. The management of uncomplicated UTIs is generally not affected by the results of blood tests because febrile cases are usually regarded as pyelonephritis and treated consequently.

Due to the nonspecific clinical presentation and increased risk of urosepsis, blood tests are generally recommended for infants aged under 3 months [13,18].

According to a Cochrane systematic review, procalcitonin is the most helpful parameter to differentiate renal involvement from lower infections and to identify renal injury in children [53,62]. Complete blood count, CRP, procalcitonin, and renal function tests are also indicated in older unwell-appearing children requiring hospitalization to monitor sepsis and exclude renal failure [60].

Uncertainty on indications for hospitalization and blood tests emerged during the first survey. After collective review of hospitalization criteria, panelists agreed on suggesting blood tests in all patients aged under 3 months or requiring hospitalization.

### 3.4. Treatment

**Statement** **10.** *Empirical antibiotic therapy is indicated in all patients presenting with fever (CT ≥ 38 °C) and urine dipstick positive for leukocyte esterase (LE) and/or nitritis and/or presence of leukocyturia and bacteriuria in a fresh urine specimen. Empirical antibiotic therapy is not indicated for asymptomatic bacteriuria (i.e., bacteriuria without fever or symptoms and without leukocyturia). When indicated, empirical therapy should be started as soon as possible within 3–4 days from fever onset. Intravenous regimens are recommended in case of sepsis, dehydration, inability to take or poor compliance to oral therapy, and should be considered for patients younger than 3 months. Intravenous therapy should be switched to oral route 24–48 h after defervescence, according to clinical conditions*.

In a febrile child with suggestive clinical signs and/or positive urine dipstick or microscopy, antibiotic treatment should be started soon after a urine specimen for culture has been collected [18]. All international guidelines agree on the indication of prompt empirical therapy when urine dipstick results are positive for both LE and nitrites [13,18,53,60,63]. Due to its high specificity, antibiotic therapy may be administered without delay also when only nitrite test results are positive [17]. When urine dipstick results are positive for LE alone, antibiotic therapy should be started only after exclusion of other sources of infection or in presence of urinary tract symptoms [13,17].

Asymptomatic bacteriuria is defined by significant presence of bacteria in urine specimen proved with culture or microscopic exam but without any signs or symptoms of infection [60,64]. This condition indicates attenuation of uropathogens by the host or colonization by avirulent bacteria that are incapable of activating a symptomatic response. Antibiotic treatment of asymptomatic bacteriuria is not indicated [60,64].

Prompt antibiotic treatment is necessary to prevent bacteremia and risk of urosepsis, which is higher in the first 3 months of life [3]. However, the role of early antibiotic treatment in preventing renal scarring is debated [65]. A clinical trial showed no significant differences between early and delayed antibiotic treatment in terms of renal scarring rates [20]. Instead, a cohort study that combined data from two previously conducted longitudinal studies showed that delay in treatment of febrile UTIs and permanent renal scarring are associated [66]. Anyway, empirical treatment should not be delayed for more than 72 h from fever onset in order to avoid acute complications [18].

Intravenous antibiotic regimens are not required in well-appearing children when similar oral alternatives are available. Clinical trials already documented the noninferiority of entirely oral antibiotic regimens, and a large Cochrane systematic review confirmed these results [57,58,59]. Antibiotic therapy should be started parenterally in complicated UTI, switching to the oral route as soon as the clinical conditions allow it or 24–48 h after defervescence [46]. Guidelines from NICE and EAU/ESPU suggest beginning with intravenous empirical treatment in all patients younger than 3 months due to the increased risk of urosepsis [13,60].

Most of the text in Statement 10 obtained sufficient agreement during the first survey. The role of urine extemporaneous microscopic analysis was initially questioned. On the basis of data from literature reporting the high sensitivity and specificity associated with detection of both bacteriuria and leukocyturia, agreement on indication for empirical antibiotic therapy in cases of positive microscopic analysis was achieved.

**Statement** **11.** *Empirical antibiotic therapy should be changed only when clinical failure occurs (defined by persistence of fever or lack of clinical improvement) regardless of susceptibility testing, with close clinical monitoring*.

When empirical antibiotic therapy is successful, fever resolves in 68% of children within the first 24 h, in 89% by 48 h, and in 92% by 72 h [67]. No clinical differences were observed between patients who took longer than 48 h to defervesce and those whose fevers responded faster to therapy. If fever persists beyond 72 h, the clinician should reevaluate the diagnosis, rule out renal abscess or other acute complications, and adjust the antibiotic therapy [68].

Italian guidelines suggest that if urine culture results show resistance to the prescribed antibiotic, but the patient’s condition is improving, treatment should be continued without change [18]. Recent observational studies reported a significant discordance between the results of in vitro susceptibility testing and the clinical outcomes observed in vivo, supporting the importance of fever resolution and clinical improvement when evaluating the effectiveness of empirical antibiotics. Discordant treatments, defined as initial antibiotics to which infecting isolates are later found not to be susceptible, may still be effective in more than half of cases [27,69].

Overall, the text in Statement 11 failed to obtain sufficient agreement during the first survey. After collective review of available established guidelines, during the second survey panelists agreed on a definition of clinical treatment failure and on the need to adjust empirical therapy only when clinical failure occurs.

**Statement** **12.** *The empirical use for treatment of amoxicillin and trimethoprim/sulfamethoxazole should be avoided because of the worldwide trend of high resistance rates among uropathogens. Suggested empirical treatments are: (1) combinations of penicillins and beta-lactamase inhibitors for patients older than 3 months affected by uncomplicated UTI; (2) third generation cephalosporins for patients older than 3 months affected by complicated UTI or presenting with risk factors for infections caused by resistant uropathogens (e.g., history of recurrent UTIs and antibiotic therapy in the previous 30 days); (3) combinations of penicillins with aminoglycosides or cephalosporins for patients younger than 3 months affected by complicated UTI. Combinations of penicillins and beta-lactamase inhibitors should be prescribed at high dosages. Patients allergic to beta-lactams should be treated with aminoglycosides. Fluoroquinolones should be reserved only for severe or non-responsive cases. Treatments of recurrent UTIs should be based on previous urine cultures and susceptibility tests*.

A large multicenter study conducted in 16 pediatric nephrology centers in 10 European countries analyzed 4745 positive urine cultures [70]. Resistance to amoxicillin for inpatients urine cultures was higher than 50% in 14/16 centers. When analyzing outpatients’ urine cultures, 8/16 centers reported rates of resistance to amoxicillin higher than 50% and 6/16 centers reported rates ranging from 21% to 50%. All centers reported that resistance to trimethoprim/sulfamethoxazole occurred in more than 21% of inpatients isolates, while resistance rates in outpatients’ isolates was higher than 21% in 8/16 centers [70]. A global meta-analysis reported rates of resistance to trimethoprim/sulfamethoxazole in pediatric UTIs caused by *E. coli* ranging from 30.2% in high-income countries and 69.6% in countries outside the Organization for Economic Co-operation and Development (OECD) [39]. In a recent review of guidelines from AAP, Mattoo et al. highlighted that the vast majority of uropathogens are susceptible to third-generation cephalosporins, and on the other hand, the authors advised against the use of amoxicillin due to the high resistance rates [68].

A recent update of guidelines from EAU/ESPU reaffirmed that, due to increasing resistance reported in pediatric UTIs, good antibiotic stewardship should guide the choice of antibiotics, considering local resistance patterns, previous urine cultures and clinical parameters [71]. According to available national data on antimicrobial-resistance patterns, guidelines from the Italian Society of Pediatric Nephrology (SINePe) recommended co-amoxiclav or ampicillin/sulbactam as first choice for empirical therapy, due to the increased prevalence of ESBL-producing uropathogens [18,72]. However, a more recent observational study reported that combinations of penicillins and beta-lactamase inhibitors, widely used in the same epidemiological context, were associated with increased risk of treatment failure [26]. Indeed, in vitro resistance to co-amoxiclav is more frequent than expected, involving up to one third of cases [26]. These findings underline how the preferential use of one antibiotic leads to the emergence of resistance.

Combinations of penicillins and beta-lactamase inhibitors may still be preferred in uncomplicated UTIs and should be administered at high dosages [3]. Despite variable resistance rates, third-generation cephalosporins appear to remain a valid first-line therapeutic option associated with protection against poor outcomes [26]. For this reason, third-generation cephalosporins should be preferred in complicated UTIs and in patients with risk factors for UTIs caused by resistant uropathogens (i.e., history of recurrent UTI, antibiotic prophylaxis and antibiotic therapy in the preceding 30 days, and urological malformations). In patients aged under 3 months, combinations of penicillins and cephalosporins or aminoglycosides should be preferred due to the increased risk of urosepsis in infants [60]. Aminoglycosides are appropriate alternatives in patients allergic to beta-lactams [73,74]. The use of quinolones in pediatric patients is controversial and should be limited to patients who are unresponsive to other antibiotics, only on the basis of susceptibility patterns [75,76]. In addition, the restriction of general use of quinolones in children in some countries and the worrying increase in resistance due to their widespread use in adults should also be taken into consideration.

Most of the text of Statement 12 obtained sufficient agreement during the first survey. Only the role of trimethoprim/sulfamethoxazole was questioned. After collective discussion on available data reporting high resistance rates for trimethoprim/sulfamethoxazole among uropathogens, experts agreed to advise against its use as empirical therapy.

**Statement** **13.** *Antibiotic therapy should be continued for at least 7–10 days in patients with febrile uncomplicated UTI and for at least 10–14 days in patients with complicated UTI. Duration of antibiotic therapy may be reduced to 5 days in case of infection limited to lower urinary tract in patients aged >3 months*.

Optimal duration of antibiotic therapy in pediatric patients affected by UTI is still controversial. New high-quality evidence from randomized clinical trials supports the effectiveness of short (5–7 days) antibiotic courses in adult patients [77,78]. Accordingly, different studies investigated the effectiveness of shorter antibiotic regimens in pediatric population. A recent observational study in children reported that 6–9 days of antibiotic treatment is as effective as a longer duration of 10 days in patients affected by febrile UTI [79]. Outcomes of even shorter courses (1–3 days) are inferior to those of longer courses [23].

Oral antibiotic therapy for at least 7–10 days is adequate for simple febrile UTI [68]. When parenteral therapy is required, after defervescence is achieved, a short course (2–4 days) of intravenous therapy followed by oral therapy for a total treatment duration of 10–14 days is as effective as a longer course (7–10 days) of intravenous therapy alone [59]. In a recent study, researchers reported that in infants aged <60 days with urosepsis, 7 days of parenteral antibiotic therapy followed by oral courses for a total duration of 14 days may be sufficient [80].

Simple cystitis may be treated with 3–5 days of antibiotics in patients aged >3 months [81].

Overall, all the text in Statement 13 obtained sufficient agreement during the first survey and was confirmed in the second survey.

**Statement** **14.** *In patients affected by complicated UTI and concomitant obstructive uropathy, temporary urinary diversion may be considered after failure of both empirical and second-line antibiotic therapies, defined as lack of clinical improvement after 72 h of an adjusted second-line therapy*.

In a recent update, the European Association of Urology suggested considering temporary urinary diversion in patients with obstructive uropathy after failure of conservative treatments [71]. In clinical practice, failure of medical treatment should be defined by a lack of clinical improvement despite at least 72 h of adequate and broad-spectrum antibiotic therapy on the basis of the results of susceptibility testing.

Uncertainty on the role and the indications of temporary urinary diversion emerged during the first survey. Pediatric surgeons involved in the panel of experts contributed to provide available evidence on the role of this procedure. Even though the urinary diversion is not frequently indicated for the treatment of pediatric UTI, this procedure might offer some advantages in selected patients with obstructive disease or other anatomical anomalies. The drainage of infected urine might avoid the progression to the formation of a renal abscess and preserve kidney function. Furthermore, the collection of infected urine might be useful for targeting the antimicrobial therapy. Therefore, agreement was achieved in the second survey.

### 3.5. Imaging

**Statement** **15.** *Renal and bladder ultrasound (RBUS) is indicated in all children, at least 2–4 weeks after a first febrile UTI in order to exclude urological anomalies. RBUS during the acute phase of infection is indicated only in case of complicated or atypical UTI, defined as sepsis, fever persisting after 72 h of adequate antibiotic therapy, oliguria, elevated plasma creatinine, or pathogens other than E. coli. Isolated dilatation of renal pelvis <10 mm is not an indication to further imaging exams. Additional radiological exams should be considered if renal hypoplasia, severe dilatation of renal pelvis, ureteral dilatation, or uroepithelial thickening occur*.

According to guidelines from NICE, RBUS is indicated in all children with UTI and aged <6 months, whereas in older children who respond well to therapy, routine US may not be needed [13]. This restrictive approach is cost-effective, but may miss a significant number of urological anomalies that are reported in 15–37% of first UTI episodes in children [21].

Most guidelines suggest performing RBUS in all patients aged <3 years with a first episode of UTI and in cases of complicated or atypical forms in older children [46]. SINePe and AAP recommend performing RBUS after at least 2 weeks from an acute episode in order to better detect renal and urinary tract anomalies [18,23]. A deferred RBUS permits more accurate interpretation of the anatomy, with less false-positive findings associated with tissue edema or endotoxin-induced dilation. Only guidelines from urologists of the EAU/ESPU suggest performing RBUS during the acute episode in all children with febrile UTI if no improvement is seen within 24 h, because 1–2% of patients with urological anomalies require prompt action (e.g., drainage) [60,82]. Other guidelines recommend RBUS during the acute episode only in cases of complicated or atypical UTI, defined as sepsis, fever persisting after 72 h of adequate antibiotic therapy, oliguria, elevated plasma creatinine, or pathogens other than *E. coli* [18,56]. Isolated dilation of renal pelvis < 10 mm should not require further radiological exams [18].

Agreement on the text of Statement 15 was obtained only after accurate definition of isolated dilatation of renal pelvis.

**Statement** **16.** *Fluoroscopic contrast voiding cystouretherography (VCUG) is the gold-standard method for the diagnosis of vesicoureteral reflux (VUR) and provides information on the anatomy of lower urinary tract. VCUG is indicated after the first episode of febrile UTI if it is caused by pathogens other than E. coli or when RBUS reveals renal hypoplasia, severe dilatation of renal pelvis, ureteral dilatation, uroepithelial thickening, or bladder abnormalities. VCUG is indicated in all second febrile UTIs. VCUG is always required before planning operative treatment of VUR*.

VCUG is the gold standard for the diagnosis and grading of VUR [3]. This technique is also useful in visualizing the anatomy of the urethra and bladder, and therefore is essential before planning operative treatment of VUR [71]. The disadvantages of this method include radiation exposure and discomfort for the patient due to bladder catheterization.

According to most guidelines, VCUG is not routinely indicated and should only be performed if RBUS reveals abnormalities suggesting urological malformations, or in other specific clinical circumstances [46]. On the contrary, recommendations updated in 2021 by EAU/ESPU highlighted that RBUS alone misses up to 33% of patients with malformations, and thus recommended imaging exams to exclude VUR in all patients with febrile UTI and aged <1 year. Two different approaches were suggested: the bottom-up method (VCUG and, if positive, DMSA scan) or the top-down method (DMSA scan and, if positive, VCUG).

Each approach might present some benefits. A recent comparative effectiveness analysis based on the RIVUR/CUTIE database found that the top-down approach reduced the need for VCUG and RBUS despite a higher risk of UTI recurrence [46]. The most reasonable approach is performing VCUG after the first episode of febrile UTI if it is caused by pathogens other than *E. coli* or when RBUS reveals renal hypoplasia, ureteral dilatation, uroepithelial thickening, or bladder abnormalities. Comparing the most important guidelines, VCUG is always indicated after recurrent episodes, defined as two or more episodes of febrile UTIs [13,46].

Most of the text in Statement 16 obtained sufficient agreement. During the first survey, indication for VCUG after all second episodes of febrile UTI was questioned. On the basis of high prevalence of VUR reported in patients with recurrent UTIs and according to established guidelines, agreement was achieved in the second survey.

**Statement** **17.** *Direct radionuclide cystography and contrast-enhanced voiding ultrasonography, when available, represent valid alternatives to VCUG for the diagnosis of VUR. Indirect radioisotopic cystography, obtained during the last phases of a MAG3 scintigraphy, has low sensitivity and specificity for diagnosis of VUR, thus is not routinely recommended*.

According to a comparison study, diagnostic correlation between direct radionuclide cystography and VCUG exceeded 85% [83,84]. Moreover, in a cohort of children with negative VCUG, Dalirani et al. observed that direct radionuclide cystography may reveal a significant number of false-negative VUR [85]. Radiation exposure seemed lower when using direct radionuclide cystography compared to VCUG; however, more recent studies reported that effective radiation dose conferred during VCUG is significantly lower than during direct isotope cystography [86].

Contrast-enhanced voiding ultrasonography does not expose children to radiation, but requires expert technicians. A systematic review reported that when compared to VCUG, contrast-enhanced voiding ultrasonography presented higher negative predictive value (87–100%) [87].

On the contrary, indirect radioisotopic cystography presents low mean sensitivity of about 54% despite good specificity up to 90% [88]. In a recent study, revision of images by experts further decreased concordance with VCUG [88].

A lack of experience with contrast-enhanced voiding ultrasonography and direct radionuclide cystography led to initial disagreement on their diagnostic role. After collective review of available literature, the role of contrast-enhanced voiding ultrasonography and direct radionuclide cystography was assessed.

**Statement** **18.** *When performing diagnostic procedures involving urinary catheterization, antibiotic prophylaxis with trimethoprim/sulfamethoxazole (2 mg/kg of trimethoprim orally in patients >6 weeks of age) or amoxicillin/clavulanic acid (50 mg/kg of amoxicillin orally) or gentamicin (2.5 mg/kg intravenous/intramuscular) immediately before the procedure is recommended for children with strongly suspected or already proven urinary abnormalities*.

Imaging procedures requiring catheterization may increase the risk of iatrogenic UTI. Given the high incidence of urologic abnormalities in children undergoing once-off urinary catheterization procedures, the development of UTIs may result in severe clinical problems. For this reason, antibiotic prophylaxis is recommended in this group of patients [89].

Studies carried out in children undergoing imaging procedures requiring catheterization have shown that the risk of iatrogenic UTI is generally low, although reported rates range widely on the basis of criteria used for the diagnosis of UTI and characteristics of study populations [90]. Rates were higher in patients with preexisting urologic abnormalities, especially high-grade reflux [90]. Considering that Gram-negative bacilli and enterococci are the most likely pathogens, the combination trimethoprim/sulfamethoxazole (2 mg/kg of trimethoprim component orally in patients > 6 weeks of age) or amoxicillin/clavulanic acid (50 mg/kg of the amoxicillin component orally) or gentamicin (2.5 mg/kg intravenous/intramuscular) immediately prior to intervention can be recommended [91,92].

Overall, all the text of Statement 18 obtained sufficient agreement during the first survey and was confirmed in the second survey.

**Statement** **19.** *Scintigraphy is not routinely recommended after the first UTI. Renal cortical scintigraphy with technetium-99m labeled dimercaptosuccinic acid (DMSA) is recommended in all children with VUR grades IV and V, at least 6 months after the febrile UTI in order to detect renal scarring*.

A DMSA scan is a useful diagnostic tool to detect both acute pyelonephritis and late renal parenchymal scarring. Guidelines from EAU/ESPU still consider DMSA scan also as part of the diagnostic approach of VUR [60]. However, it is an expensive technique that exposes the patient to radiation, and it usually does not affect acute clinical management. Moreover, different studies reported that DMSA scans have limited ability to replace VCUG in the diagnosis of VUR [93].

Indications for DMSA scans vary considerably among guidelines, probably due to their unclear role in clinical decisions. Guidelines from NICE suggest performing DMSA scans in atypical (defined as seriously ill, poor urine flow, abdominal or bladder mass, raised creatinine, septicemia, failure to respond to treatment with suitable antibiotics within 48 h, or infections caused by non-*E. coli* pathogens) or recurrent UTIs in children aged <3 years and only in recurrent infections in children aged >3 years [13]. Italian guidelines recommended DMSA scans only in patients with VUR grades IV and V, because it is the most important risk factor associated with renal scarring [94]. Optimal timing to detect renal scarring with DMSA scan is about 4–6 months after an acute episode [13,94].

Overall, all the text of Statement 19 obtained sufficient agreement during the first survey and was confirmed in the second survey.

### 3.6. Prophylaxis

**Statement** **20.** *Long-term antibiotic prophylaxis seems to have no effect on the risk of UTI recurrence, and it is not routinely indicated. Antibiotic prophylaxis may be considered until performing VCUG, when it is indicated, in children with history of recurrent UTIs (i.e., ≥3 episodes/year) or with VUR grade IV–V. Oral amoxicillin/clavulanic acid or third generation cephalosporins represent valid options for long-term prophylaxis. There is not enough evidence to define which are the most proper molecules, dosages and duration for long-term antibiotic prophylaxis. Doses from one third to one half of those administered during the acute infection are generally considered suitable for long-term prophylaxis*.

Despite being studied extensively, the effectiveness of antimicrobial prophylaxis in the prevention of UTI recurrence is still controversial. Conflicting results from recent literature have been attributed to significant differences in study designs, including patient inclusion and exclusion criteria.

When considering all pediatric patients after a first episode of UTI, long-term antibiotic prophylaxis seems to have little or no effect on the risk of recurrence. A multicenter, randomized, controlled trial conducted on 338 children affected by a first episode of acute pyelonephritis observed that antibiotic prophylaxis did not reduce the rate of recurrent UTIs after 12 months in children without VUR or with low-grade VUR [95]. A large meta-analysis reported that long-term antibiotics may reduce the risk of repeat symptomatic UTI in children who have had one or more previous UTIs, with or without VUR, but the supposed benefit was small and not statistically significant [96].

Moreover, a probable increased risk of UTI caused by resistant pathogens in children on prophylaxis has been reported [96]. In populations of patients with known risk factors for recurrent UTIs, long-term antibiotic prophylaxis may play a role. The RIVUR trial reported that prophylaxis with trimethoprim/sulfamethoxazole reduced the risk of recurrence by 50% in children with VUR. Similar results were reported by another placebo-controlled, double-blind trial conducted on children with at least grade III VUR [97]. However, the benefits of prophylaxis seem limited to children with high-grade VUR. Randomized controlled trials conducted on children with low- or mixed-grade VUR failed to demonstrate the effectiveness of antibiotic prophylaxis [98,99,100]. Combined results of the RIVUR and the Careful Urinary Tract Infection Evaluation (CUTIE) studies revealed that children with high-grade VUR and bladder–bowel dysfunction (BBD) may exhibit the greatest benefit from antimicrobial prophylaxis [96,101]. However, even when considering only patients with concomitant VUR, evidence on the effectiveness of long-term prophylaxis in preventing recurrence of UTIs is not exhaustive, as observed by Williams et al. in a systematic review [102].

No study has demonstrated any effect of antimicrobial prophylaxis in preventing renal scarring [103]. In light of controversial available evidence, a selective approach for long-term antibiotic prophylaxis seems more appropriate to obtain real benefits balancing the increased risk of antimicrobial resistance. The American Urological Association, EAU/ESPU, and Swedish and Italian Society of Pediatric Nephrology recommend antibiotic prophylaxis only on the basis of a combination of patient age, severity of VUR, history of recurrent UTIs, and preexisting renal scarring [18,60,104,105]. Accordingly, antibiotic prophylaxis may be considered until performing VCUG, when it is indicated, in children with history of recurrent UTIs (≥3 episodes/year) or with VUR grade IV–V.

Italian guidelines suggest amoxicillin/clavulanic acid as first choice for antibiotic prophylaxis, while nitrofurantoin and trimethoprim/sulfamethoxazole are preferred in recommendations from EAU/ESPU. Due to the already high resistance rates shown by uropathogens against trimethoprim/sulfamethoxazole and the inactivity of nitrofurantoin against *Proteus* spp., amoxicillin/clavulanic acid seems a better option for the selected patients for whom it is recommended [18,60]. Oral cephalosporins represent the second choice in both guidelines [18,60]. There is insufficient evidence to recommend a specific dose; however, traditionally, the dose used for prophylaxis has been from one third to one half of the treatment dose, given once per day [18].

A deep discussion on antibiotic prophylaxis in pediatric UTIs was required among the experts. High-quality evidence available from recent literature was collectively reviewed and commented. Panelists agreed on underlining that there is still not enough evidence on the effectiveness of long-term antibiotic prophylaxis and on which are the most proper molecules, dosages, and duration. The second survey concluded that antibiotic prophylaxis may be considered only in patients with specific risk factors.

**Statement** **21.** *There is insufficient evidence on the effectiveness of cranberry preparations and probiotic in preventing relapses of UTI. Modifiable risk factors for the occurrence of UTI are phimosis, constipation, bladder-bowel dysfunctions and low daily water intake. In children with recurrent UTIs or urinary tract malformations, urine culture is indicated only when fever and/or symptoms or clinical signs of UTI occur*.

The efficacy of cranberry juice for the prevention of recurrent pediatric UTI is debated [18]. In a recent study, it was found that cranberry extract prevents adhesion of *E. coli* to uroepithelial cells, suggesting the effect of this metabolites in recurrent UTI [106]. In a review of eight clinical trials, cranberry products modestly reduced the incidence of recurrent UTI in children with normal urinary anatomy [107]. In a randomized, controlled trial on children with recurrent UTIs but no or minor urologic malformations, administration of cranberry products did not significantly reduce the number of children who experienced a recurrence of UTI, but it was effective in reducing the actual number of recurrences and related antimicrobial use [108].

Different studies about probiotic effectiveness in preventing of recurrent UTI were proposed based on their effect on the production of antimicrobial products, competition with uropathogens, and occupation of the epithelial space to prevent adherence of uropathogenic bacteria [109,110]. A recent Cochrane review reported that no significant benefit was demonstrated for probiotics compared with placebo or no treatment in preventing recurrent UTI [111].

Bowel and bladder dysfunction is a combination of lower urinary tract symptoms and bowel disorders, including constipation and/or encopresis, in patients with no known neurological abnormality [112,113]. Overactive bladder that results from detrusor overactivity and voiding postponement that occurs when a patient delays urination are the most common lower urinary tract symptoms. Others include underactive bladder caused by detrusor underactivity, dysfunctional voiding caused by habitual contraction of the bladder sphincter and pelvic floor, and bladder-neck dysfunction, which refers to delayed or impaired bladder opening that results in reduced urine flow despite increased bladder pressure. Constipation causes urinary retention as a result of compression of the bladder and elongation of the urethra by fecal retention. In addition, some children with constipation present renal pelvic dilation even in the absence of anatomical abnormalities or infection [114]. All these conditions may result in postvoid urine retention, leading to increase bacterial replication and risk of recurrent UTI [115]. Correction of lower urinary tract symptoms and treatment of constipation are important to decrease the rate of UTI recurrence [116,117]. Therefore, the main international guidelines recommend exclusion of bowel and bladder dysfunction and lower urinary tract symptoms in any children with febrile and/or recurrent UTI [13,60].

Increased oral fluid helps flush bacteria from the bladder, prompts frequent urination, and alleviates constipation, and therefore it may be reasonable that it plays a role in preventing UTI. However, the lack of enough adequately powered and robust RCTs highlights the need for further research on the effectiveness of this intervention for UTI prevention [118].

Phimosis is another known risk factor of recurrent UTI. A recent study showed that the use of steroid cream for physiological phimosis is associated with a decreased risk of recurrent UTIs in uncircumcised male infants with a normal renal ultrasound [119].

According to most guidelines, screening and treatment for asymptomatic bacteriuria should always be discouraged, even in children with recurrent UTIs or urinary tract malformations [60]. Therefore, urine culture should be considered only when fever and/or symptoms or clinical signs of UTI occur.

Overall, all the text of Statement 21 obtained sufficient agreement during the first survey and was confirmed in the second survey.

## 4. Discussion

This study shows that there are ongoing controversies on the management of pediatric UTI, mainly related to prevalence of antibiotic resistance, importance of extemporaneous urinalysis, appropriate methods for collection of urine samples, indications for blood tests, use of antibiotics, and approach to complicated cases.

The emergence of antibiotic resistance is an unavoidable phenomenon closely correlated with the use of antibiotics themselves. Limited knowledge was observed on the prevalence of XDR uropathogens and resistance to trimethoprim/sulfamethoxazole. To contain the emergence of resistance, every effort to reduce and rationalize antibiotic consumption must be made. Our study highlighted discrepancies on empirical antibiotic therapy in suspected UTI, as well as on the awareness that discordant empirical treatments may still be effective in more than half of pediatric UTIs, and a differentiated empirical approach is recommended only for patients presenting risk factors for treatment failure. A deep discussion was required for agreement on the indications for antibiotic prophylaxis, and the experts concluded on a limitation of its use for the absence of evidence on its effectiveness. An increased use of antibiotic stewardship can be greatly effective in this regard.

Also, lack of knowledge was observed on the importance of microscopic examination of urine on extemporaneous urinalysis and the use of a bag to collect urine samples for diagnosis. This issue could lead to overprescription of antibiotics, and our review of the literature presented the appropriate approach. Similarly, our study highlighted the importance of sharing prescriptive appropriateness paths for blood-examination requests in cases of suspected UTI.

Furthermore, indications on second-line approaches (i.e., temporary urinary diversion, fluoroscopic VCUG, contrast-enhanced voiding ultrasonography, and direct radionuclide cystography) appeared poorly known by primary care pediatricians. With a view to integrated hospital-territory assistance, this limit must be overcome, because it is necessary that even professionals working in primary care know when to prescribe additional diagnostic tests for their patients.

Through the Delphi method, in our study the participants discussed the statements and agreement was reached after an active discussion in some cases. It should be noted that the participants in the project came from different clinical contexts, i.e., they were pediatricians, infectious diseases specialists, pediatric urologists, and pediatric surgeons. For this reason, the results achieved demonstrate the usefulness of the Delphi method for the selection of good practices and constitute the basis of an evidence-based approach. The findings obtained can establish the basis for educational interventions that aim to optimize the use of antibiotics and prescription of diagnostic exams in pediatric patients with UTI. Limitations of the study include that it was an opinion-based survey and agreement was reached at a collegial meeting. On the other hand, the lack of pediatric studies on several UTI-related topics did not permit use of the GRADE.

This consensus document aimed to respond to issues that are still little addressed, with the ambition to fill current shortcomings. The specific statements developed are intended to guide the health-care professional in practice to ensure a better and standardized management of the neonatal and pediatric patient. Box 1 summarizes the 21 statements.

Box 1 **Management of urinary tract infections in pediatric age.**

**Epidemiology**

**Statement** **1.** *E. coli is the most common pathogen in pediatric UTIs accounting for more than 70% of all cases, followed by Klebsiella spp., Enterobacter spp., and Proteus spp. Pseudomonas aeruginosa is uncommon in community-acquired pediatric UTIs but it is associated with more severe infections. Up to 30% of pediatric patients experience a recurrence after the first episode of UTI*.

**Statement** **2.** *Prevalence of pathogens resistant to antibiotic therapy varies widely in different geographical areas. The main risk factors for UTIs caused by resistant pathogens include urinary tract anatomical or functional abnormalities, long-term antibiotic prophylaxis, and exposure to antibiotics during previous 30 days*.

**Statement** **3.** *Prevalence of uropathogens resistant to combinations of penicillins and beta-lactamase inhibitors and third generation cephalosporins in pediatric population is increasing worldwide. Prevalence of ESBL-producing pathogens in pediatric UTIs is globally increasing. Prevalence of MDR pathogens is low but increasing, whereas prevalence of extensively drug-resistant (XDR) pathogens is still low and stable*.

**Diagnosis**

**Statement** **4.** *Diagnosis of UTI should be considered in all children with fever (CT > 38 °C) without clear localization. In children aged <3 months, an episode of UTI may occur with vomiting, irritability or lethargy even without a fever. Lack of fever in children aged <3 months does not correlate with the severity of UTI. The most frequent symptoms in older children with UTI are dysuria, urinary urgency, increased voiding frequency, new-onset incontinence, abdominal pain and low back pain. The detection of malodorous urine is not specific enough to diagnose UTI*.

**Statement** **5.** *Rapid extemporaneous urinalysis (dipstick) is indicated in all children with fever (CT > 38 °C) without clear localization and in those that have symptoms and clinical signs compatible with UTI. The presence of leukocyte esterase and nitrite combined shows elevated sensitivity and specificity for the diagnosis of UTI. Isolated presence of nitrites has high specificity but low sensitivity for diagnosis of UTI. Isolated presence of leukocyte esterase has high sensitivity but low specificity for diagnosis of UTI. Absence of nitrites and leukocyte esterase makes diagnosis of UTI highly unlikely. The presence of bacteriuria and leukocyte on microscopic examination of urine on extemporaneous urinalysis is associated with high specificity and sensitivity for diagnosis of UTI*.

**Statement** **6.** *Positive urine culture is necessary to confirm the diagnosis of UTI. Urine culture with antibiogram is indicated in case of positive nitrite and/or leukocyte esterase or leukocyturia and bacteriuria. Urine culture is not indicated in absence of both nitrite and leukocyte esterase*.

**Statement** **7.** *A urine sample suitable for culture should always be collected before starting empirical antibiotic therapy. The collection of samples for urine culture in children in good clinical condition should be performed by clean catch mid-stream void or transurethral bladder catheterization; for those in compromised general conditions should be performed by transurethral bladder catheterization. The use of a sterile bag for the collection of urine samples for culture may be acceptable only if the bag is placed for less than 20 min and considering significant only bacterial growth > 100,000 CFU/mL. Urine culture should be regarded as positive only if a single pathogenic species is isolated*.

**Management**

**Statement** **8.** *Hospitalization is suggested for patients aged under 3 months, critically ill children requiring intravenous therapy (i.e., vomiting, dehydration, sepsis), failure of oral therapy (i.e., persistence of fever after 72 h of adequate antibiotics), or poor compliance to oral therapy*.

**Statement** **9.** *Blood tests are not routinely needed in children affected by febrile UTI. Complete and differential blood count, C-reactive protein (CRP), procalcitonin, and kidney function tests are recommended in children aged under 3 months and in all cases requiring hospitalization*.

**Treatment**

**Statement** **10.** *Empirical antibiotic therapy is indicated in all patients presenting with fever (CT ≥ 38 °C) and urine dipstick positive for leukocyte esterase (LE) and/or nitritis and/or presence of leukocyturia and bacteriuria in a fresh urine specimen. Empirical antibiotic therapy is not indicated for asymptomatic bacteriuria (i.e., bacteriuria without fever or symptoms and without leukocyturia). When indicated, empirical therapy should be started as soon as possible within 3–4 days from fever onset. Intravenous regimens are recommended in case of sepsis, dehydration, inability to take or poor compliance to oral therapy, and should be considered for patients younger than 3 months. Intravenous therapy should be switched to oral route 24–48 h after defervescence, according to clinical conditions*.

**Statement** **11.** *Empirical antibiotic therapy should be changed only when clinical failure occurs (defined by persistence of fever or lack of clinical improvement) regardless of susceptibility testing, with close clinical monitoring*.

**Statement** **12.** *The empirical use for treatment of amoxicillin and trimethoprim/sulfamethoxazole should be avoided because of the worldwide trend of high resistance rates among uropathogens. Suggested empirical treatments are: (1) combinations of penicillins and beta-lactamase inhibitors for patients older than 3 months affected by uncomplicated UTI; (2) third generation cephalosporins for patients older than 3 months affected by complicated UTI or presenting with risk factors for infections caused by resistant uropathogens (e.g., history of recurrent UTIs and antibiotic therapy in the previous 30 days); (3) combinations of penicillins with aminoglycosides or cephalosporins for patients younger than 3 months affected by complicated UTI. Combinations of penicillins and beta-lactamase inhibitors should be prescribed at high dosages. Patients allergic to beta-lactams should be treated with aminoglycosides. Fluoroquinolones should be reserved only for severe or non-responsive cases. Treatments of recurrent UTIs should be based on previous urine cultures and susceptibility tests*.

**Statement** **13.** *Antibiotic therapy should be continued for at least 7–10 days in patients with febrile uncomplicated UTI and for at least 10–14 days in patients with complicated UTI. Duration of antibiotic therapy may be reduced to 5 days in case of infection limited to lower urinary tract in patients aged >3 months*.

**Statement** **14.** *In patients affected by complicated UTI and concomitant obstructive uropathy, temporary urinary diversion may be considered after failure of both empirical and second-line antibiotic therapies, defined as lack of clinical improvement after 72 h of an adjusted second-line therapy*.

**Imaging**

**Statement** **15.** *Renal and bladder ultrasound (RBUS) is indicated in all children, at least 2–4 weeks after a first febrile UTI in order to exclude urological anomalies. RBUS during the acute phase of infection is indicated only in case of complicated or atypical UTI, defined as sepsis, fever persisting after 72 h of adequate antibiotic therapy, oliguria, elevated plasma creatinine, or pathogens other than E. coli. Isolated dilatation of renal pelvis < 10 mm is not an indication to further imaging exams. Additional radiological exams should be considered if renal hypoplasia, severe dilatation of renal pelvis, ureteral dilatation, or uroepithelial thickening occur*.

**Statement** **16.** *Fluoroscopic contrast voiding cystouretherography (VCUG) is the gold-standard method for the diagnosis of vesicoureteral reflux (VUR) and provides information on the anatomy of lower urinary tract. VCUG is indicated after the first episode of febrile UTI if it is caused by pathogens other than E. coli or when RBUS reveals renal hypoplasia, severe dilatation of renal pelvis, ureteral dilatation, uroepithelial thickening, or bladder abnormalities. VCUG is indicated in all second febrile UTIs. VCUG is always required before planning operative treatment of VUR*.

**Statement** **17.** *Direct radionuclide cystography and contrast-enhanced voiding ultrasonography, when available, represent valid alternatives to VCUG for the diagnosis of VUR. Indirect radioisotopic cystography, obtained during the last phases of a MAG3 scintigraphy, has low sensitivity and specificity for diagnosis of VUR, thus is not routinely recommended*.

**Statement** **18.** *When performing diagnostic procedures involving urinary catheterization, antibiotic prophylaxis with trimethoprim/sulfamethoxazole (2 mg/kg of trimethoprim orally in patients > 6 weeks of age) or amoxicillin/clavulanic acid (50 mg/kg of amoxicillin orally) or gentamicin (2.5 mg/kg intravenous/intramuscular) immediately before the procedure is recommended for children with strongly suspected or already proven urinary abnormalities*.

**Statement** **19.** *Scintigraphy is not routinely recommended after the first UTI. Renal cortical scintigraphy with technetium-99m labeled dimercaptosuccinic acid (DMSA) is recommended in all children with VUR grades IV and V, at least 6 months after the febrile UTI in order to detect renal scarring*.

**Prophylaxis**

**Statement** **20.** *Long-term antibiotic prophylaxis seems to have no effect on the risk of UTI recurrence, and it is not routinely indicated. Antibiotic prophylaxis may be considered until performing VCUG, when it is indicated, in children with history of recurrent UTIs (i.e., ≥3 episodes/year) or with VUR grade IV–V. Oral amoxicillin/clavulanic acid or third generation cephalosporins represent valid options for long-term prophylaxis. There is not enough evidence to define which are the most proper molecules, dosages and duration for long-term antibiotic prophylaxis. Doses from one third to one half of those administered during the acute infection are generally considered suitable for long-term prophylaxis*.

**Statement** **21.** *There is insufficient evidence on the effectiveness of cranberry preparations and probiotic in preventing relapses of UTI. Modifiable risk factors for the occurrence of UTI are phimosis, constipation, bladder-bowel dysfunctions and low daily water intake. In children with recurrent UTIs or urinary tract malformations, urine culture is indicated only when fever and/or symptoms or clinical signs of UTI occur*.

## 5. Conclusions

This consensus provides clear and shared indications on UTI management in pediatric age, based on the most updated literature. This work represents, in our opinion, the most complete and up-to-date collection of statements on practices to follow in pediatric UTI, in order to guide physicians in the management of the patient, standardizing approaches, and avoiding abuse and misuse of antibiotics. Undoubtedly, more randomized and controlled trials are needed in the pediatric population to better define the best therapeutic management in cases with antimicrobial resistance and the real usefulness of long-term antibiotic prophylaxis.

## Data Availability

All the data are included in the manuscript.

## Supplementary Materials

The following supporting information can be downloaded at: https://www.mdpi.com/article/10.3390/antibiotics11081122/s1, Supplementary Materials S1: Questionnaire with statements. Supplementary Materials S2: Results of the first and second surveys.
