# Supplementary material for: Management of Pediatric Urinary Tract Infections: A Delphi Study
Source: Antibiotics (Basel). 2022 Aug 18;11(8):1122. doi: 10.3390/antibiotics11081122 (PMC9404756; doi:10.3390/antibiotics11081122)
Supplement: Supplementary file 1 [file antibiotics-11-01122-s001.zip › antibiotics-1820999-supplementary-1.pdf]

Supplementary material 1

Study questionnaire with statements

SURVEY	AGREEMENT (%)
1.Escherichia coli is the most common pathogen in pediatric UTIs accounting for more than 70% of all cases, followed by Klebsiella spp, Enterobacter spp, and Proteus spp.	100,0
2. Pseudomonas aeruginosa in uncommon in community acquired pediatric UTIs but it is associated with more severe infections.	100,0
3. Up to 30% of pediatric patients experience a recurrence after the first episode of UTI.	95,1
4. Prevalence of pathogens resistant to antibiotic therapy varies widely in different geographical areas.	97,6
5. The main risk factors for UTIs caused by resistant pathogens include urinary tract anatomical or functional abnormalities	95,1
6. The main risk factors for UTIs caused by resistant pathogens include long-term antibiotic prophylaxis	97,6
7. The main risk factors for UTIs caused by resistant pathogens include exposure to antibiotics during previous 30 days	95,1
8. Prevalence of uropathogens resistant to combinations of penicillins and beta-lactamase inhibitors in pediatric population is increasing worldwide.	100,0
9. Prevalence of uropathogens resistant to third generation cephalosporins in pediatric population is increasing worldwide.	97,6
10. Prevalence of extended spectrum beta-lactamase (ESBL)-producing pathogens in pediatric UTIs is globally increasing.	97,6
11. Prevalence of multidrug resistant (MDR) pathogens is globally low but increasing.	97,6
12. Prevalence of extensively drug-resistant (XDR) pathogens is still low and stable.	90,2
13. Diagnosis of UTI should be considered in all children with fever (CT > 38°C) without clear localization.	100,0
14. In children aged < 3 months, an episode of UTI may occur with vomiting, irritability or lethargy even without a fever.	100,0
15. Lack of fever in children aged < 3 months does not correlate with the severity of UTI.	97,6
16. The most frequent symptoms in older children with UTI are dysuria, urgent need to urinate, pollachiuria, abdominal pain and low back pain.	100,0
17. The detection of malodorous urine is not specific enough to diagnose UTI.	100,0

18. Rapid extemporaneous urinalysis (dipstick) is indicated in all children with fever (> 38°C) without clear localization and in those that have symptoms and clinical signs compatible with UTI.	100,0
19. The presence of leukocytic esterase and nitrite combined shows elevated sensitivity and specificity for the diagnosis of UTI.	97,6
20. Isolated presence of nitrites has high specificity but low sensitivity for diagnosis of UTI.	95,1
21. Isolated presence of leukocyte esterase has high sensitivity but low specificity for diagnosis of UTI.	90,2
22. Absence of nitrites and leukocyte esterase makes diagnosis of UTI highly unlikely.	97,6
23.The presence of bacteriuria and leukocyte on microscopic examination of urine on extemporaneous urinalysis is associated with high specificity and sensitivity for diagnosis of UTI.	92,7
24. Urine culture with antibiogram is indicated in case of positive nitrite and/or leukocyte esterase or leukocyturia and bacteriuria.	97,6
25. Urine culture is not indicated in absence of both nitrite and leukocyte esterase.	97,6
26. Positive urine culture is necessary to confirm the diagnosis of UTI.	97,6
27. A urine sample suitable for culture should always be collected before starting empirical antibiotic therapy.	100,0
28. The collection of samples for urine culture in children in good clinical condition should be performed by clean catch mid-stream void or bladder catheterization	97,6
29. The collection of samples for urine culture in children in compromised general conditions should be performed by transurethral bladder catheterization.	100,0
30. The use of a sterile bag for the collection of urine samples for culture may be acceptable only if the bag is placed for less than 20 minutes and considering significant only bacterial growth >100,000 CFU/ml.	92,7
31. Urine culture should be regarded as positive only if a single pathogenic specie is isolated	87,8
32. Hospitalization is suggested for critically ill children requiring intravenous therapy (e.g. vomiting, dehydration, sepsis), failure of oral therapy (persistence of fever after 72 hours of adequate antibiotics), or poor compliance to oral therapy.	100,0
33. Hospitalization is recommended for children with UTIs aged under 3 months	95,1
34. Blood tests are not routinely needed in children affected by febrile UTI.	95,1
35. Complete and differential blood count, C-reactive protein (CRP), procalcitonin, and kidney function tests are recommended in children aged under 3 months.	95,1
36. Complete and differential blood count, C-reactive protein (CRP), procalcitonin, and kidney function tests are recommended in children over 3 months old in all cases requiring hospitalization.	100,0

37. Empirical antibiotic therapy is indicated in all patients presenting with fever (≥38°C) and urine dipstick positive for leucocyte esterase (LE) and/or nitritis	100,0
38. Empirical antibiotic therapy is indicated in all patients presenting with fever (≥38°C) and presence of leucocyturia and bacteriuria in a fresh urine specimen.	95,1
39. Empirical antibiotic therapy is not indicated for asymptomatic bacteriuria (i.e., bacteriuria without fever or symptoms and without leucocyturia).	100,0
40. When indicated, empirical therapy should be started as soon as possible within 3-4 days from fever onset.	100,0
41. Intravenous regimens are recommended in case of sepsis, dehydration, inability to take or poor compliance to oral therapy, and should be considered for patients younger than 3 months.	100,0
42. Intravenous therapy should be switched to oral route 24-48 hours after defervescence, according to clinical conditions.	100,0
43. Empirical antibiotic therapy should be adjusted only when clinical failure occurs, defined by persistence of fever or lack of clinical improvement	95,1
44. Empirical antibiotic therapy should be adjusted only when clinical failure occurs, on the basis of susceptibility testing.	100,0
45. The empirical use of amoxicillin should be avoided because of high resistance rates among uropathogens.	97,6
46. Suggested empirical treatments are combinations of penicillins and beta-lactamase inhibitors for patients older than 3 months affected by uncomplicated UTI	97,6
47. Suggested empirical treatments are third generation cephalosporins for patients older than 3 months affected by complicated UTI	95,1
48. Suggested empirical treatments are third generation cephalosporins for patients older than 3 months affected by complicated UTI or presenting with risk factors for infections caused by resistant uropathogens (e.g., history of recurrent UTIs and antibiotic therapy in the previous 30 days)	95,1
49. Suggested empirical treatments are combinations of penicillins with aminoglycosides or cephalosporins for patients younger than 3 months affected by complicated UTI.	100,0
50. Patients allergic to beta-lactams should be treated with aminoglycosides.	90,2
51. The empirical use of trimetoprim/sulfamethoxazole should be avoided because of high resistance rates among uropathogens.	92,7
52. Fluoroquinolones should be reserved only for severe or non-responsive cases.	97,6
53. Treatments of recurrent UTIs should be based on previous urine cultures and susceptibility tests.	95,1
54. Combinations of penicillins and beta-lactamase inhibitors should be prescribed at high dosages.	92,7

55. Antibiotic therapy should be continued for at least 7-10 days in patients with febrile uncomplicated UTI	100,0
56. Antibiotic therapy should be continued for at least 10-14 days in patients with complicated UTI (es. urosepsis).	100,0
57. Duration of antibiotic therapy may be reduced to 5 days in case of infection limited to lower urinary tract in patients aged > 3 months.	92,7
58. In patients affected by complicated UTI and concomitant obstructive uropathy, temporary urinary diversion may be considered after failure of both empirical and second-line antibiotic therapies, defined as lack of clinical improvement after 72 hours of adjusted second therapy.	90,2
59. Renal and bladder ultrasound (RBUS) during the acute phase of infection is indicated only in case of complicated or atypical UTI, defined as sepsis, fever persisting after 72 hours of adequate antibiotic therapy, oliguria, elevated plasma creatinine, or pathogens other than E. coli.	97,6
60. Renal and bladder ultrasound (RBUS) is indicated in all children, at least 2-4 weeks after a first febrile UTI in order to exclude urological anomalies.	92,7
61. Isolated dilatation of renal pelvis < 10 mm is not an indication to further imaging exams.	97,6
62. Fluoroscopic contrast voiding cistouretherography (VCUG) is the gold-standard method for the diagnosis of vesicoureteral reflux (VUR) and provides information on the anatomy of lower urinary tract.	100,0
63. VCUG is indicated after the first episode of febrile UTI if it is caused by pathogens other than E. coli or when RBUS reveals renal hypoplasia, severe dilatation of renal pelvis, ureteral dilatation, uroepithelial thickening, or bladder abnormalities. VCUG is indicated in all second febrile UTIs.	100,0
VCUG is indicated after the first episode of febrile UTI if it is caused by pathogens other than E. coli or when RBUS reveals renal hypoplasia, severe dilatation of renal pelvis, ureteral dilatation, uroepithelial thickening, or bladder abnormalities. VCUG is indicated in all second febrile UTIs.	
65. Contrast-enhanced voiding ultrasonography and direct radionuclide cystography, when available, represent valid alternatives to VCUG for the diagnosis of VUR.	95,1
66. VCUG is always required before planning operative treatment of VUR.	100,0
67. Indirect radioisotopic cystography, obtained during the last phases of a MAG3 scintigraphy, has low sensitivity and specificity for diagnosis of VUR, thus is not routinely recommended.	97,6
68. When performing diagnostic procedures involving urinary catheterization, antibiotic prophylaxis is recommended for children with strongly suspected or already proven urinary abnormalities.	95,1
69. When performing diagnostic procedures involving urinary catheterization, antibiotic prophylaxis with trimethoprim/sulfamethoxazole (2 mg/kg of trimethoprim p.o. in patients >6 weeks of age) or amoxicillin/clavulanic acid (50 mg/kg of	85,4

amoxicillin) or gentamicin (2.5 mg/kg i.v./i.m.) immediately before the procedure is recommended.	
70. Scintigraphy is not routinely recommended after the first UTI.	97,6
71. Renal cortical scintigraphy with technetium-99m labeled dimercaptosuccinic acid (DMSA) is recommended in all children with VUR grades IV and V, at least 6 months after the febrile UTI in order to detect renal scarring.	95,1
72. The role of long-term antibiotic prophylaxis has been questioned by several clinical studies, it seems to have no effect on the risk of recurrence, and it is not routinely indicated.	97,6
73. Antibiotic prophylaxis may be considered until performing VCUG, when it is indicated, in children with history of recurrent UTIs (≥ 3 episodes/year) or with VUR grade IV-V.	97,6
74. When prophylaxis is indicated, amoxicillin/clavulanic acid or third generation oral cephalosporins represent valid options for long-term prophylaxis.	92,7
74. When prophylaxis is indicated, amoxicillin/clavulanic acid or third generation oral cephalosporins represent valid options for long-term prophylaxis.	
75. There is not enough evidence to define which are the most proper molecules, dosages and duration for long-term antibiotic prophylaxis.	95,1
76. Doses from one third to one half of those administered during the acute infection are generally considered suitable for long-term prophylaxis.	100,0
77. There is insufficient evidence on the effectiveness of cranberry preparations in preventing relapses of UTI.	97,6
78. There is insufficient evidence on the effectiveness of probiotics in preventing relapses of UTI.	95,1
79. Modifiable risk factors for the occurrence of UTI are phimosis, constipation, bladder-bowel disfunctions and low daily water intake.	100,0
80. In children with recurrent UTIs or urinary tract malformations, urine culture is indicated only when fever and/or symptoms or clinical signs of UTI occur.	97,6
