# Supplementary material for: Management of Pediatric Urinary Tract Infections: A Delphi Study
Source: Antibiotics (Basel). 2022 Aug 18;11(8):1122. doi: 10.3390/antibiotics11081122 (PMC9404756; doi:10.3390/antibiotics11081122)
Supplement: Supplementary file 1 [file antibiotics-11-01122-s001.zip › antibiotics-1820999-supplementary-2.pdf]

EPIDEMIOLOGY

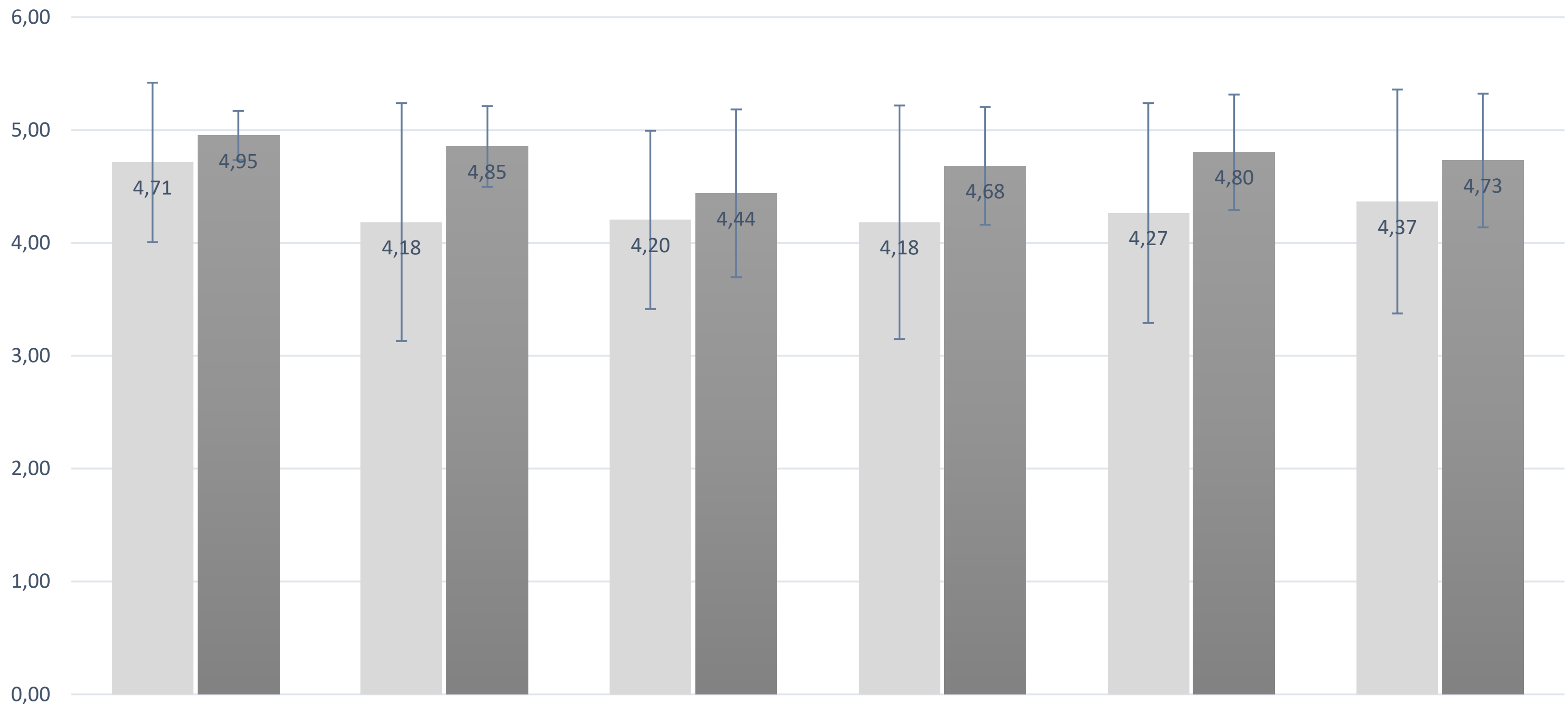

1.Escherichia coli is the most common pathogen in pediatric UTIs accounting for more than 70% of all cases, followed by Klebsiella spp, Enterobacter spp, and Proteus spp.

2. Pseudomonas aeruginosa in uncommon in community acquired pediatric UTIs but it is associated with more severe infections.

3. Up to 30% of pediatric patients experience a recurrence after the first episode of UTI.

4. Prevalence of pathogens resistant to antibiotic therapy varies widely in different geographical areas.

5. The main risk factors for UTIs caused by resistant pathogens include urinary tract anatomical or functional abnormalities

6. The main risk factors for UTIs caused by resistant pathogens include long-term antibiotic prophylaxis

**First survey (%)**	**95.9**	**83.7**	**85.7**	**81.6**	**77.6**	**83.7**
**Second survey (%)**	**100.0**	**100.0**	**95.1**	**97.6**	**95.1**	**97.6**

EPIDEMIOLOGY

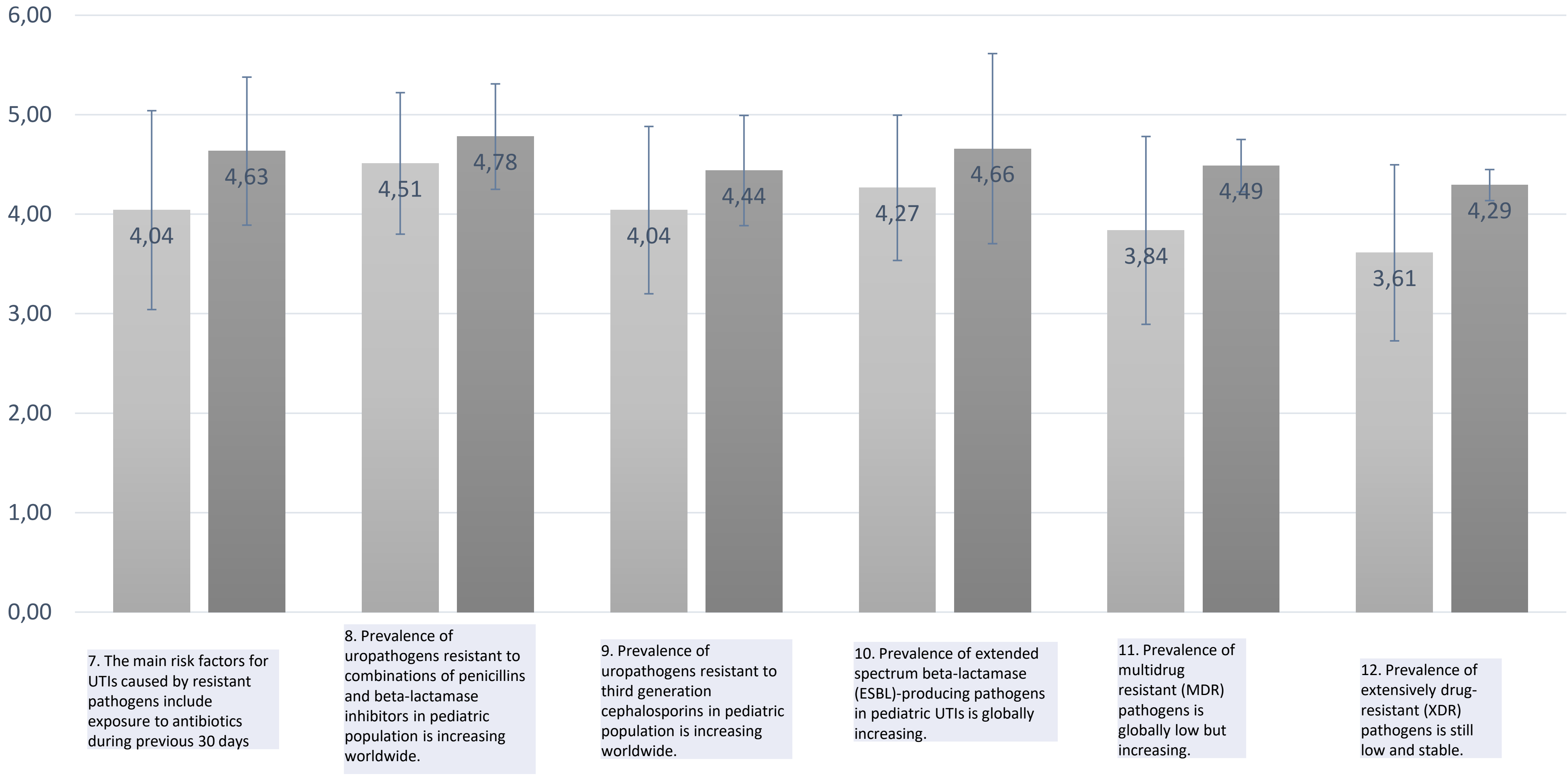

**First survey (%)**	**79.6**	**98.0**	**79.6**	**93.9**	**81.6**	**61.2**
**Second survey (%)**	**95.1**	**100.0**	**97.6**	**97.6**	**97.6**	**90.2**

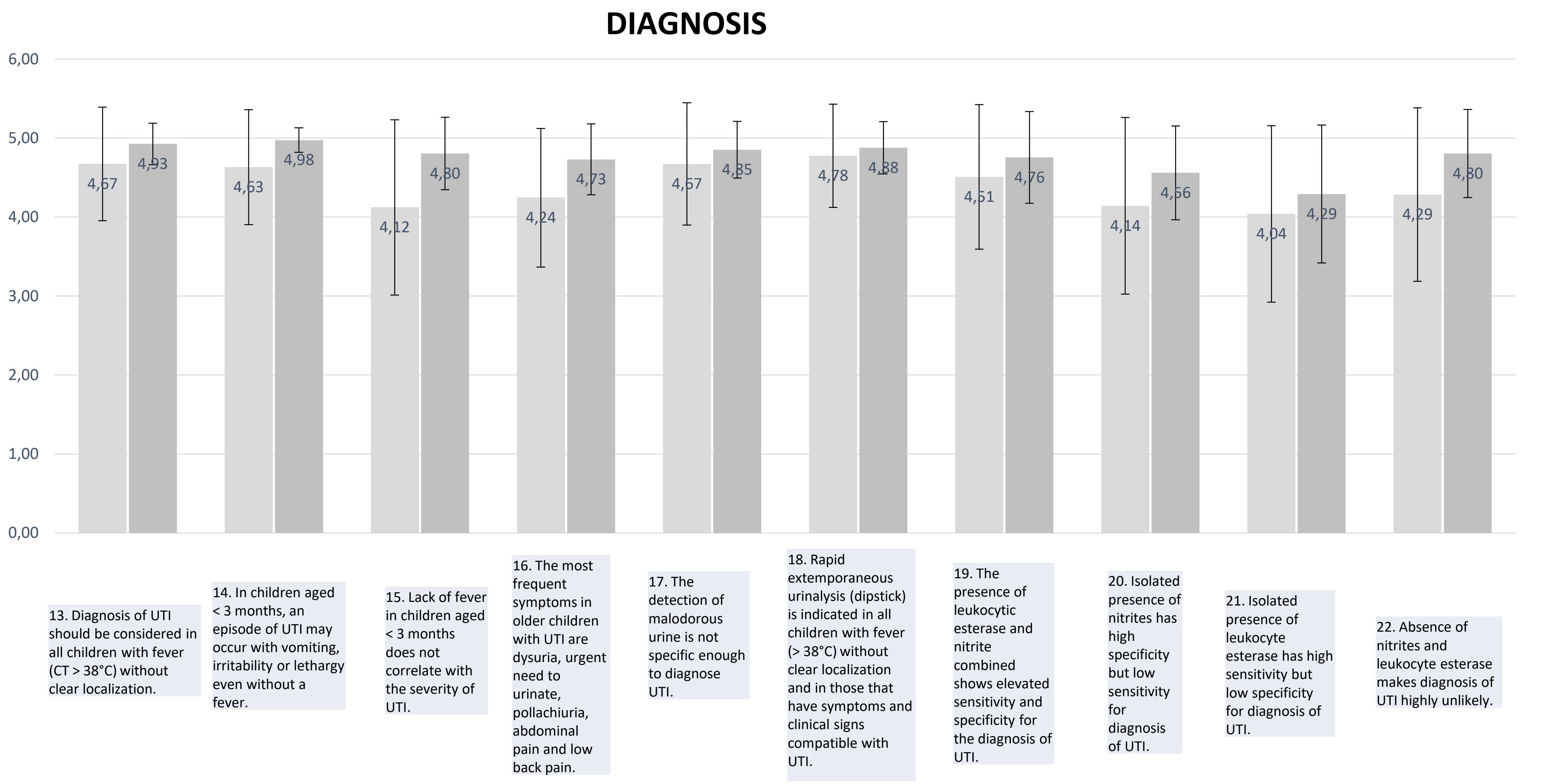

	13	14	15	16	17	18	19	20	21	22
**First survey (%)**	93.9	95.9	81.6	87.8	95.9	98.0	89.8	75.5	79.6	83.7
**Second survey (%)**	100.0	100.0	97.6	100.0	100.0	100.0	97.6	95.1	90.2	97.6

DIAGNOSIS

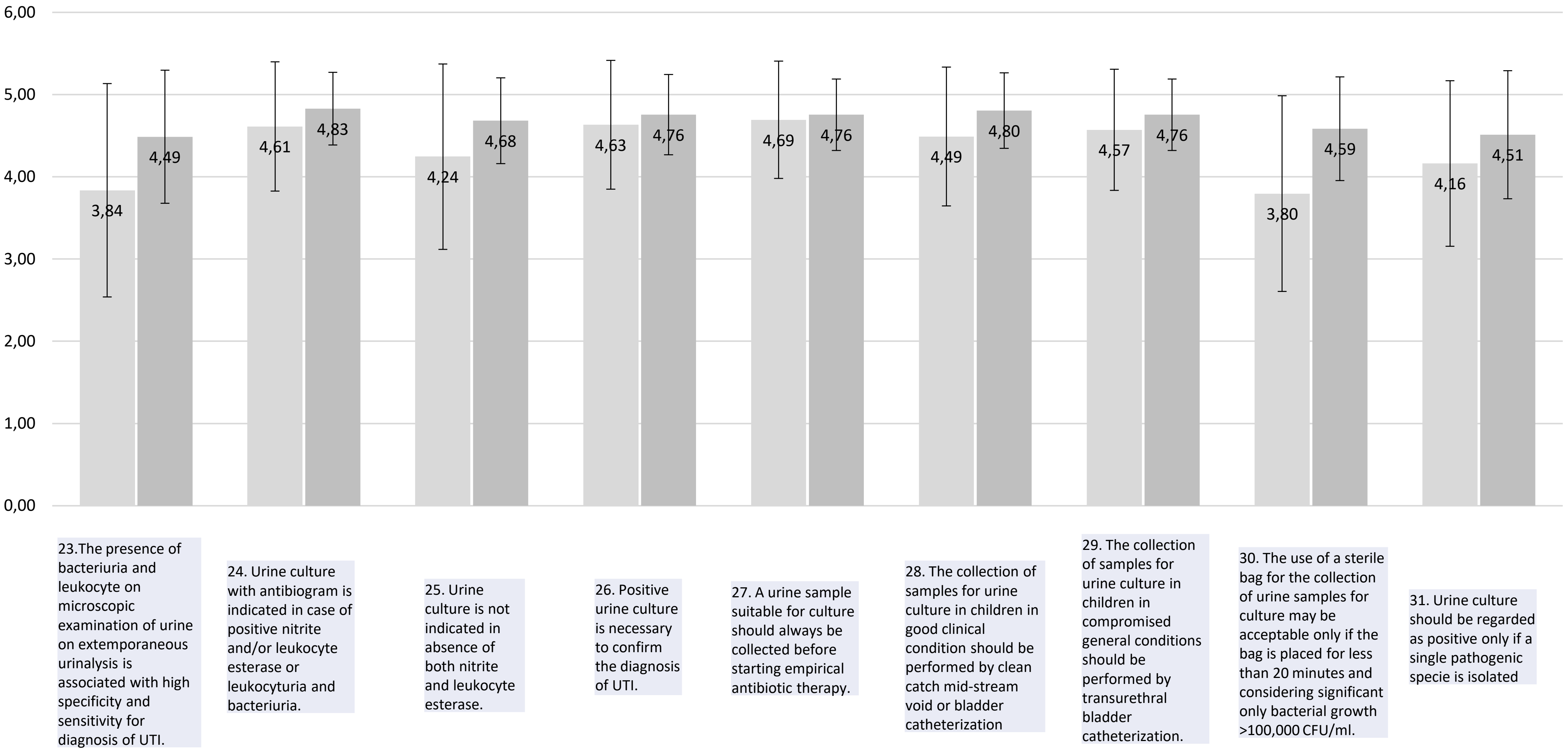

	23	24	25	26	27	28	29	30	31
**First survey (%)**	65.3	91.8	85.7	95.9	95.9	91.8	95.9	65.3	79.6
**Second survey (%)**	92.7	97.6	97.6	97.6	100.0	97.6	100.0	92.7	87.8

MANAGEMENT

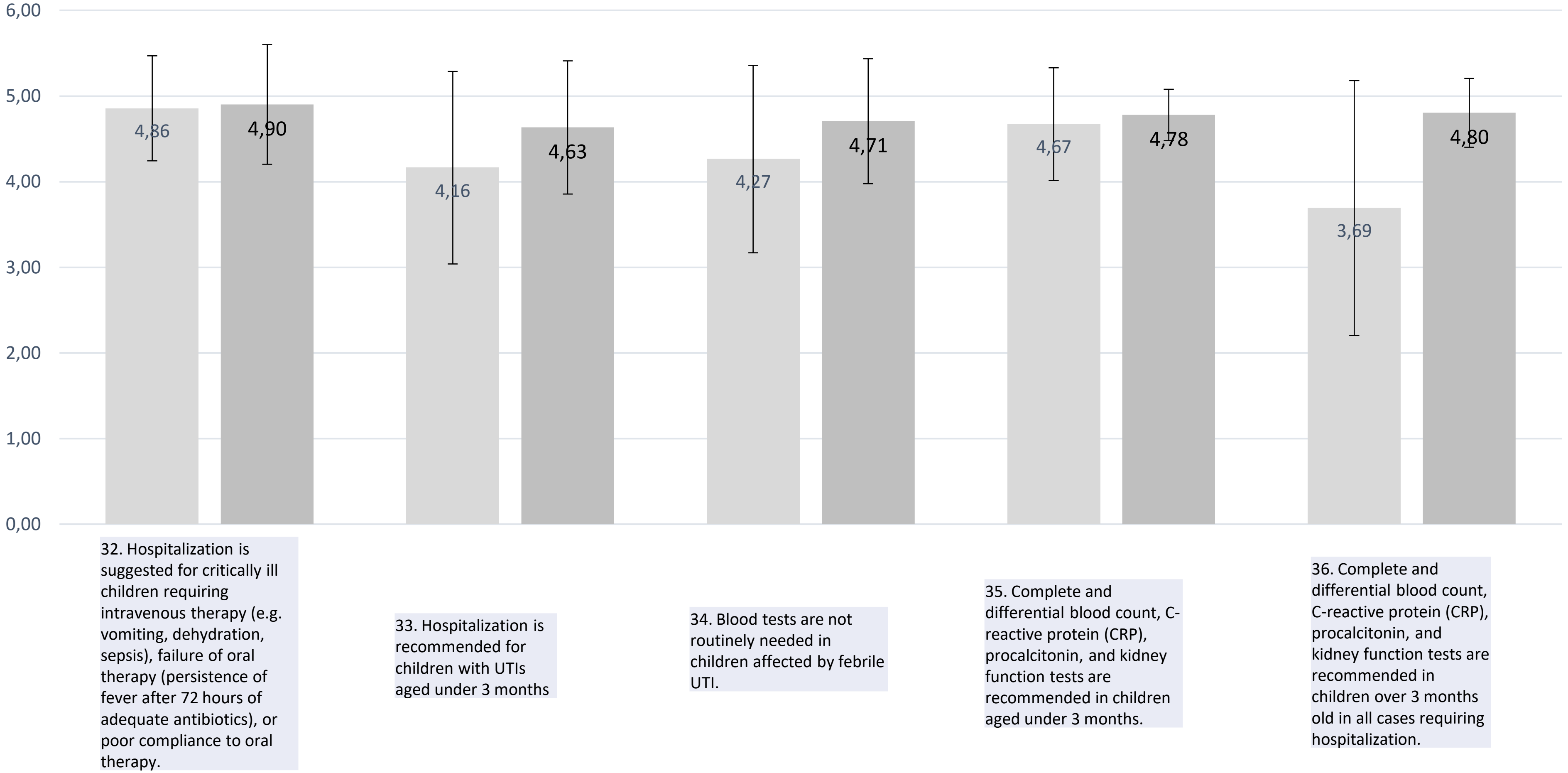

**First survey (%)**	98.0	79.6	83.7	93.9	69.4
**Second survey (%)**	100.0	95.1	95.1	95.1	100.0

TREATMENT

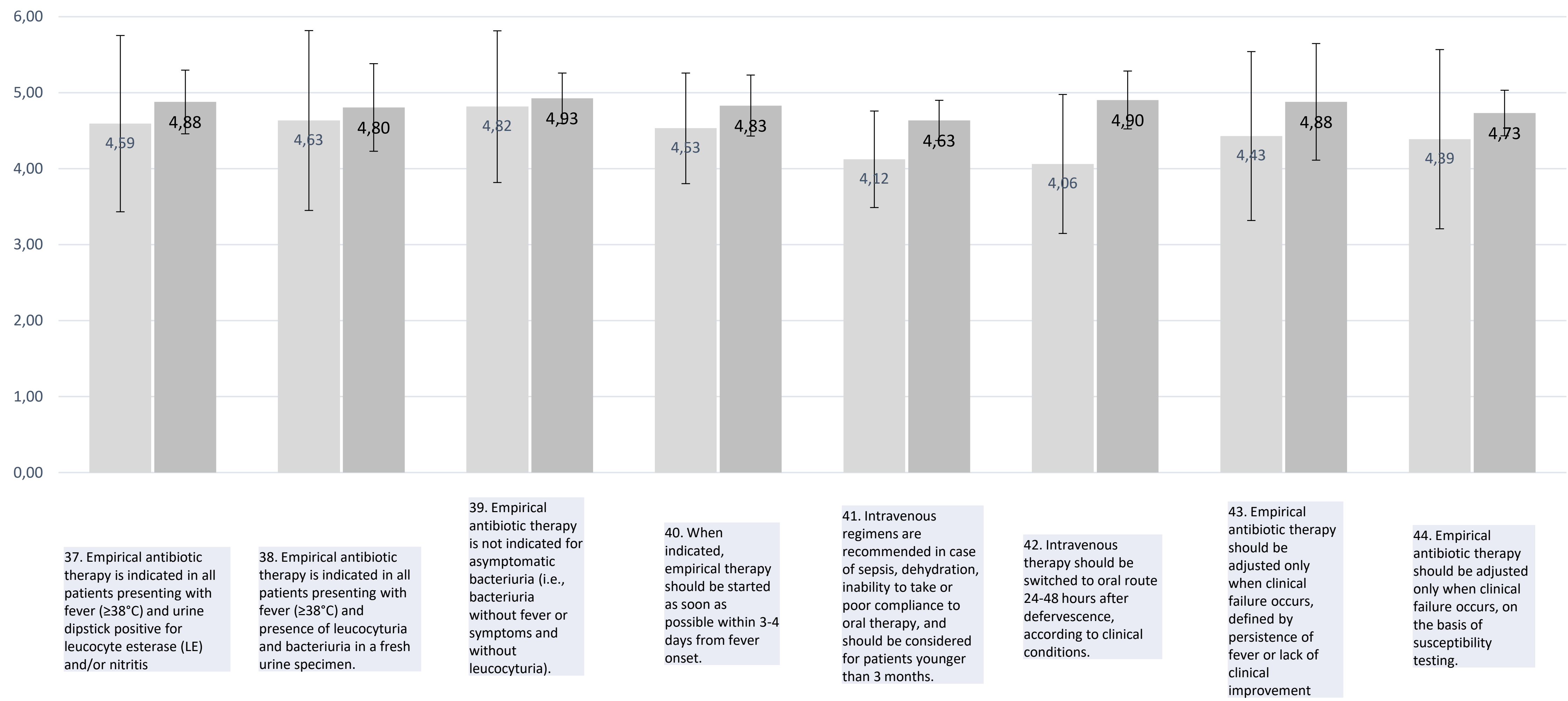

**First survey (%)**	79.6	69.4	93.9	95.9	98.0	91.8	77.6	69.4
**Second survey (%)**	100.0	95.1	100.0	100.0	100.0	100.0	95.1	100.0

TREATMENT

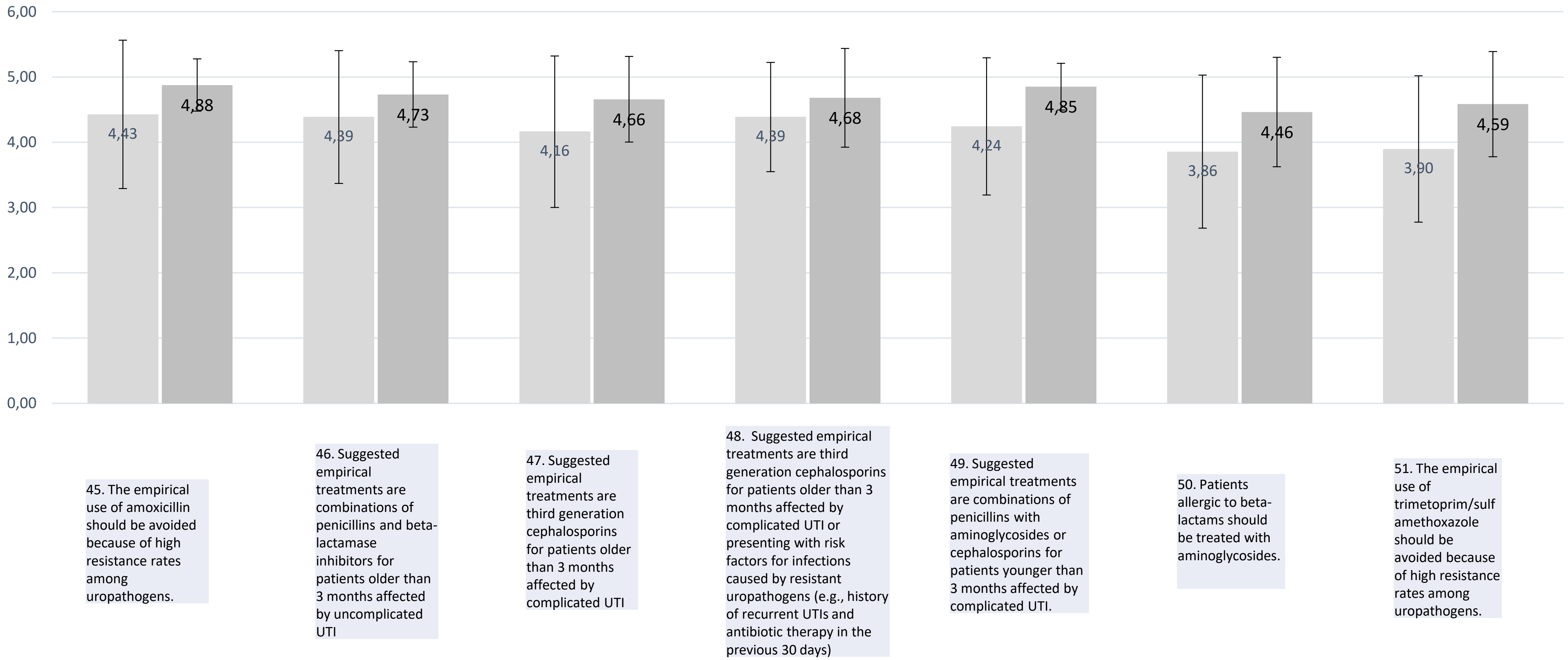

**First survey (%)**	**83.7**	**85.7**	**77.6**	**85.7**	**81.6**	**75.5**	**69.4**
**Second survey (%)**	**97.6**	**97.6**	**95.1**	**95.1**	**100.0**	**90.2**	**92.7**

TREATMENT

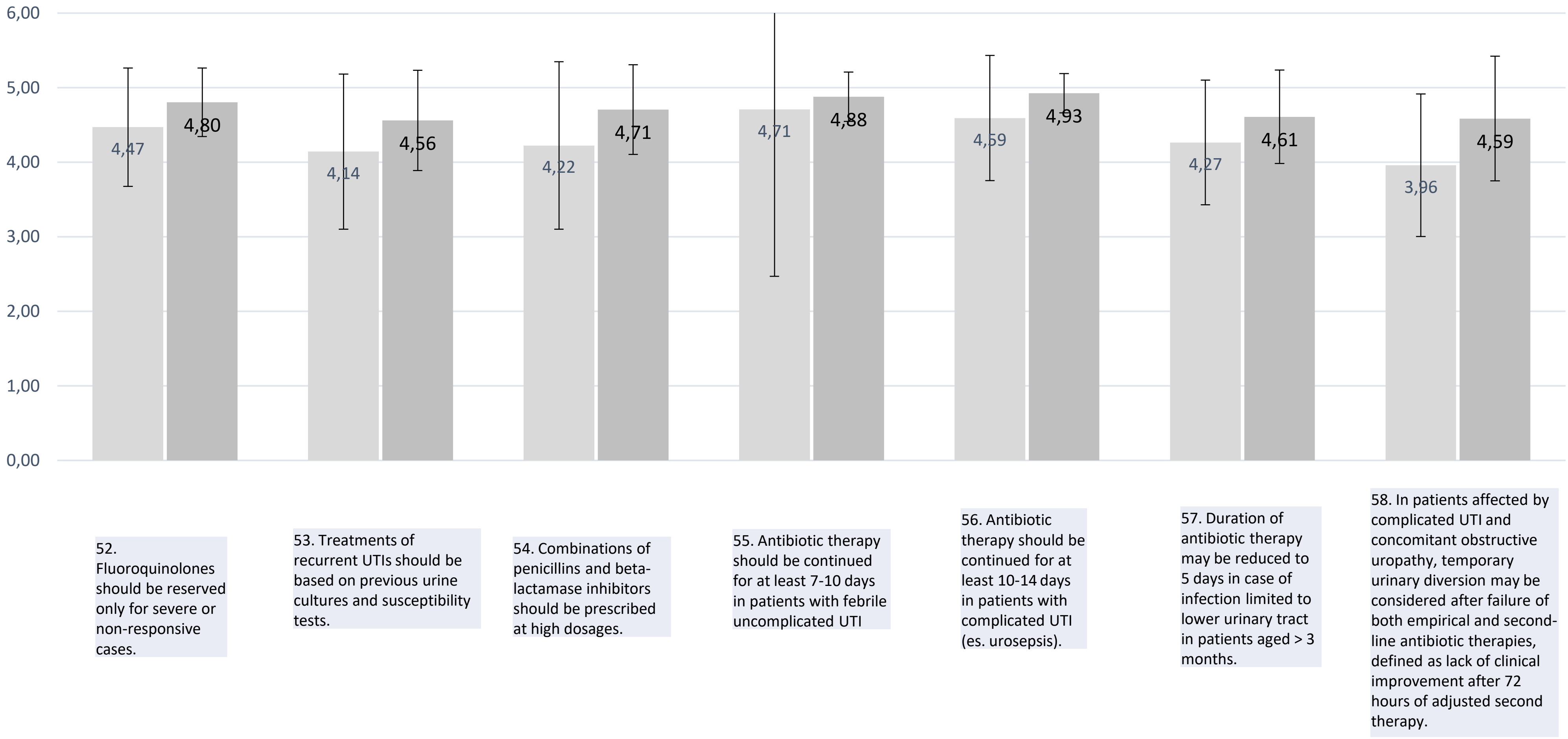

**First survey (%)**	91.8	79.6	81.6	97.1	91.8	85.7	69.4
**CSecond survey (%)**	97.6	95.1	92.7	100.0	100.0	92.7	90.2

IMAGING

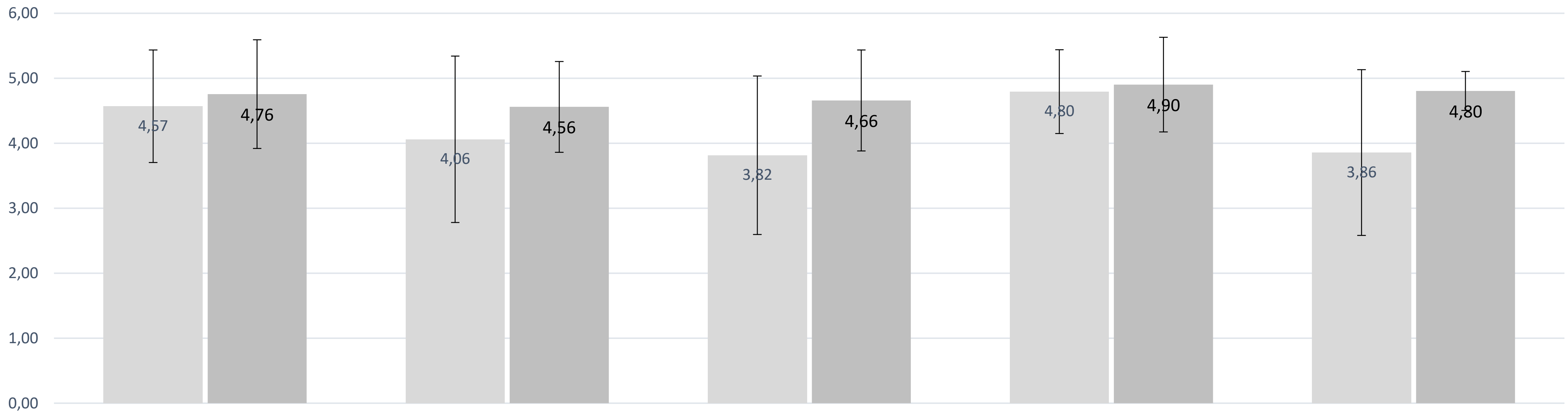

59. Renal and bladder ultrasound (RBUS) during the acute phase of infection is indicated only in case of complicated or atypical UTI, defined as sepsis, fever persisting after 72 hours of adequate antibiotic therapy, oliguria, elevated plasma creatinine, or pathogens other than E. coli.

60. Renal and bladder ultrasound (RBUS) is indicated in all children, at least 2-4 weeks after a first febrile UTI in order to exclude urological anomalies.

61. Isolated dilatation of renal pelvis < 10 mm is not an indication to further imaging exams.

62. Fluoroscopic contrast voiding cistouretherography (VCUG) is the gold-standard method for the diagnosis of vesicoureteral reflux (VUR) and provides information on the anatomy of lower urinary tract.

63. VCUG is indicated after the first episode of febrile UTI if it is caused by pathogens other than E. coli or when RBUS reveals renal hypoplasia, severe dilatation of renal pelvis, ureteral dilatation, uroepithelial thickening, or bladder abnormalities. VCUG is indicated in all second febrile UTIs.

**Prima survey (%)**	**93.9**	**79.6**	**67.3**	**98.0**	**67.3**
**Seconda survey (%)**	**97.6**	**92.7**	**97.6**	**100.0**	**100.0**

IMAGING

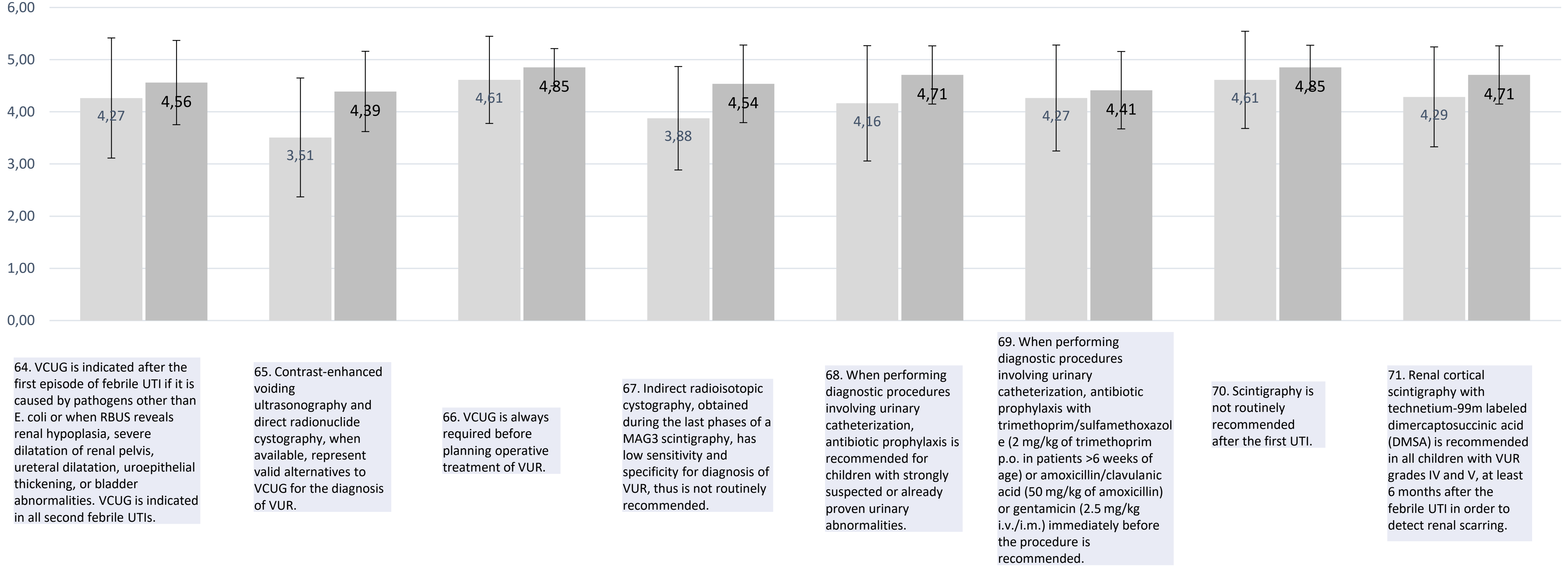

83.7	51.0	91.8	73.5	79.6	81.6	93.9	83.7
90.2	95.1	100.0	97.6	95.1	85.4	97.6	95.1

PROPHYLAXIS

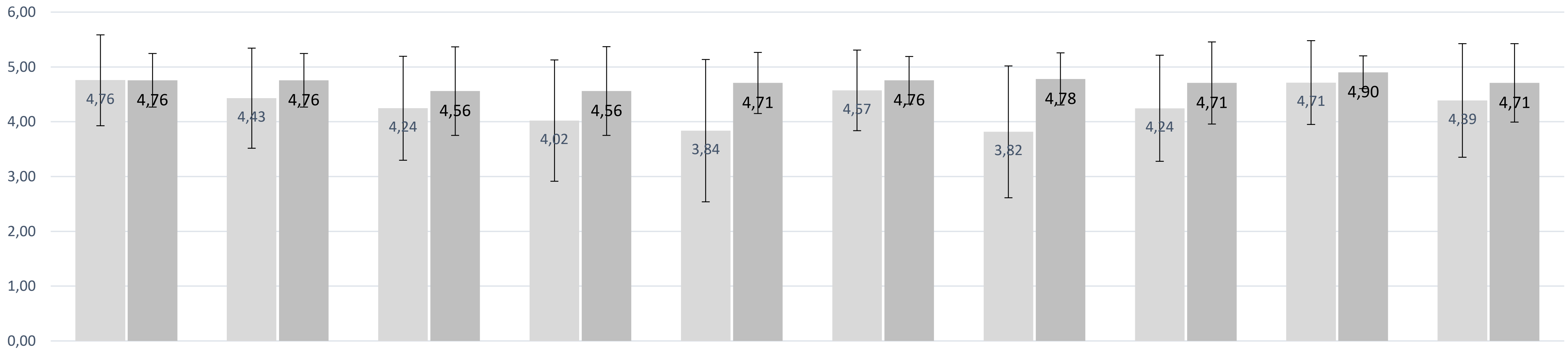

72. The role of long-term antibiotic prophylaxis has been questioned by several clinical studies, it seems to have no effect on the risk of recurrence, and it is not routinely indicated.

73. Antibiotic prophylaxis may be considered until performing VCUG, when it is indicated, in children with history of recurrent UTIs (≥ 3 episodes/year) or with VUR grade IV-V.

74. When prophylaxis is indicated, amoxicillin/clavulanic acid or third generation oral cephalosporins represent valid options for long-term prophylaxis.

74. When prophylaxis is indicated, amoxicillin/clavulanic acid or third generation oral cephalosporins represent valid options for long-term prophylaxis.

75. There is not enough evidence to define which are the most proper molecules, dosages and duration for long-term antibiotic prophylaxis.

76. Doses from one third to one half of those administered during the acute infection are generally considered suitable for long-term prophylaxis.

77. There is insufficient evidence on the effectiveness of cranberry preparations in preventing relapses of UTI.

78. There is insufficient evidence on the effectiveness of probiotics in preventing relapses of UTI.

79. Modifiable risk factors for the occurrence of UTI are phimosis, constipation, bladder-bowel disfunctions and low daily water intake.

80. In children with recurrent UTIs or urinary tract malformations, urine culture is indicated only when fever and/or symptoms or clinical signs of UTI occur.

95.9	81.6	81.6	65.3	67.3	95.9	75.5	87.8	95.9	85.7
97.6	97.6	92.7	92.7	95.1	100.0	97.6	95.1	100.0	97.6
